# Comprehensive Metabolic and Taxonomic Reconstruction of an Ancient Microbial Mat From the McMurdo Ice Shelf (Antarctica) by Integrating Genetic, Metaproteomic and Lipid Biomarker Analyses

**DOI:** 10.3389/fmicb.2022.799360

**Published:** 2022-04-12

**Authors:** María Ángeles Lezcano, Laura Sánchez-García, Antonio Quesada, Daniel Carrizo, Miguel Ángel Fernández-Martínez, Erika Cavalcante-Silva, Víctor Parro

**Affiliations:** ^1^Centro de Astrobiología (CAB), CSIC-INTA, Carretera de Ajalvir, Madrid, Spain; ^2^Departamento de Biología, C. Darwin 2, Universidad Autónoma de Madrid, Madrid, Spain; ^3^Department of Natural Resource Sciences, McGill University, Sainte-Anne de Bellevue, QC, Canada

**Keywords:** microbial mat communities, microbial metabolism, DNA metabarcoding, metaproteomics, lipid biomarkers, McMurdo Ice Shelf, Antarctica

## Abstract

Paleobiological reconstructions based on molecular fossils may be limited by degradation processes causing differential preservation of biomolecules, the distinct taxonomic specificity of each biomolecule type, and analytical biases. Here, we combined the analysis of DNA, proteins and lipid biomarkers using 16S and 18S rRNA gene metabarcoding, metaproteomics and lipid analysis to reconstruct the taxonomic composition and metabolisms of a desiccated microbial mat from the McMurdo Ice Shelf (MIS) (Antarctica) dated ~1,000 years BP. The different lability, taxonomic resolution and analytical bias of each biomolecule type led to a distinct microbial community profile. DNA analysis showed selective preservation of DNA remnants from the most resistant taxa (e.g., spore-formers). In contrast, the proteins profile revealed microorganisms missed by DNA sequencing, such as *Cyanobacteria*, and showed a microbial composition similar to fresh microbial mats in the MIS. Lipid hydrocarbons also confirmed *Cyanobacteria* and suggested the presence of mosses or vascular plant remnants from a period in Antarctica when the climate was warmer (e.g., Mid-Miocene or Eocene). The combined analysis of the three biomolecule types also revealed diverse metabolisms that operated in the microbial mat before desiccation: oxygenic and anoxygenic photosynthesis, nitrogen fixation, nitrification, denitrification, sulfur reduction and oxidation, and methanogenesis. Therefore, the joint analysis of DNA, proteins and lipids resulted in a powerful approach that improved taxonomic and metabolic reconstructions overcoming information gaps derived from using individual biomolecules types.

## Introduction

Fundamental questions in paleobiology and paleoecology depend on the accurate characterization of ancient samples, in which molecular fossils are the primary source of information (Cutler et al., 1999). However, the reliability of paleoreconstructions is limited by *post-mortem* modifications of biological remains (Cutler et al., 1999; Gomes et al., 2020), which may alter the record of taxa and lead to biased paleoreconstructions (i.e., differences between the preserved fossil record and the original biological community). Therefore, the strength of taphonomic processes altering biological signatures following death (Farmer and Des Marais, 1999), and the preservation capacity of molecular remains, ultimately determines the accuracy of the biological paleoreconstruction. DNA has been extensively used in samples so far confined to the past 1 million years (Willerslev et al., 2004; Willerslev and Cooper, 2005; Pedersen et al., 2015; Leonardi et al., 2017), and offers a high taxonomic specificity. Compared to DNA, proteins show greater resistance over time thus allowing recovery sample information further back in time (to ~3.8 million years (Rybczynski et al., 2013; Demarchi et al., 2016), or even more than 65 million years in dinosaur bones (e.g., Muyzer et al., 1992; Asara et al., 2007), albeit with some scepticism (e.g., Pevzner et al., 2008; Buckley et al., 2017). In contrast to DNA and proteins, lipids have a less taxonomic resolution but are more resistant and persistent in the environment even for billions of years (Brocks and Summons, 2003; Vinnichenko et al., 2020), so far becoming important biomolecules for environmental paleoreconstructions beyond the Mid-Pliocene.

Despite the different taxonomic resolution and preservation of biomolecules, most paleobiological reconstructions have been focused on the analysis of a single biomolecule, either DNA (Pedersen et al., 2015; Ellegaard et al., 2020), proteins (Cappellini et al., 2012; Mackie et al., 2017) or lipids (Schinteie and Brocks, 2017), limiting a comprehensive reconstruction of the past biological composition and ecological scenario of a sample. Therefore, multiproxy approaches based on the analysis of different biomolecules has recently been claimed to address complex biological questions (Cappellini et al., 2018), such as paleoecological reconstructions.

Most of the history of life on Earth has been dominated by microorganisms, some being part of microbial mats. Microbial mats are vertically stratified assemblages of microorganisms acting as self-sustaining microecosystems (Almela et al., 2019) as a result of the varied metabolisms occurring across mat layers (Van Gemerden, 1993; De los Rios et al., 2004; Prieto-Barajas et al., 2018). When a waterbody dries out (e.g., lake, pond or stream), the microbial mats that inhabit the aquatic system also dry out and may be preserved for centuries and even millennia, thus becoming relict samples that serve as ecological legacies (Moorhead et al., 1999). Therefore, ancient microbial mats represent ideal target samples for the evaluation of the paleobiological reconstruction capacity of different biomolecules due to their microbial diversity and plethora of metabolisms.

**Figure 1:**
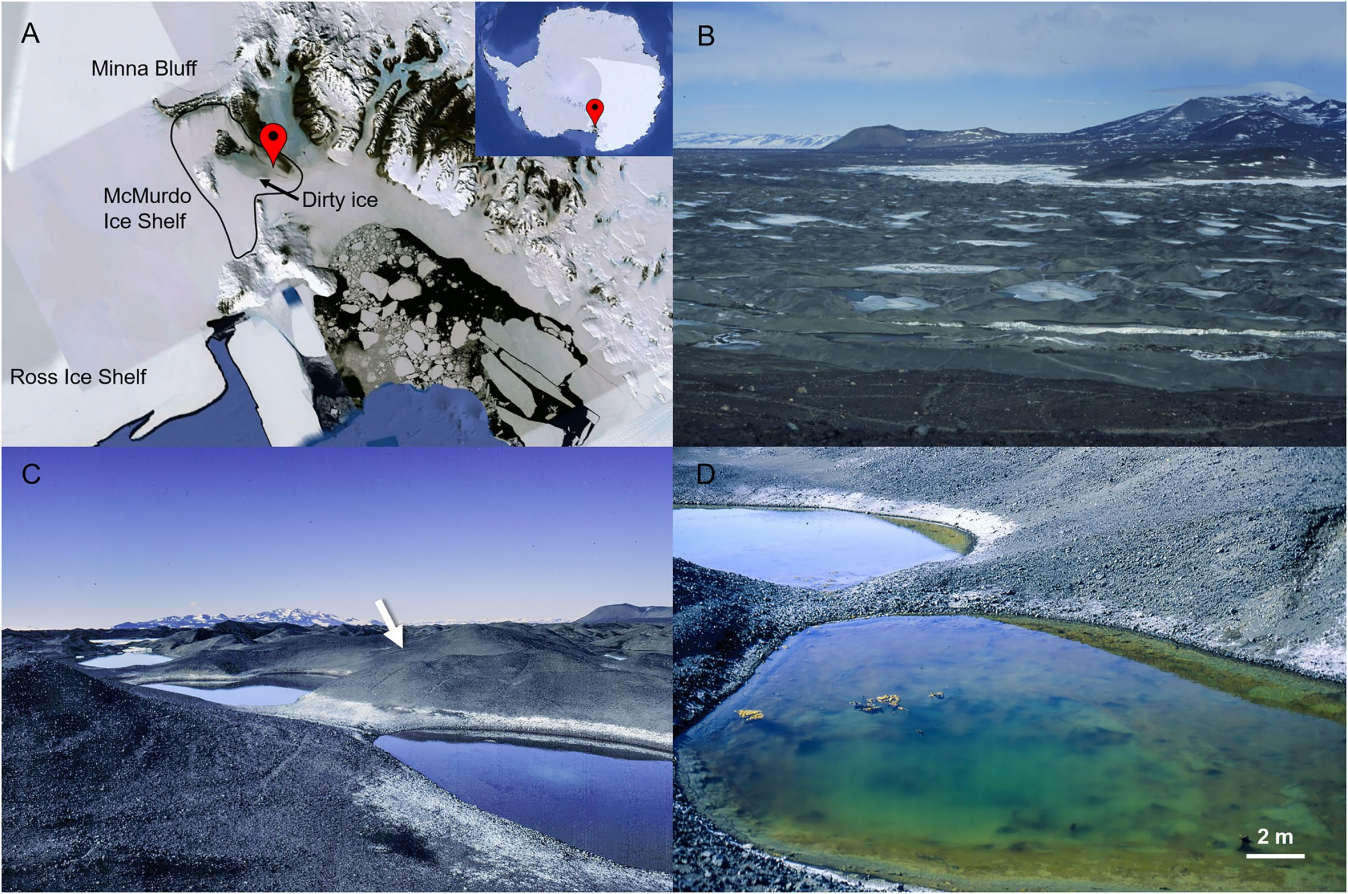
McMurdo Ice Shelf (MIS) close to the coast of Bratina Island. In **(A)**, geographic location of the MIS in Antarctica (image of Google Earth). In **(B)**, a view of the MIS from the top of Bratina Island showing the debris coated-ice of the surface (“dirty ice”) and numerous seasonal meltwater ponds (some ice-capped). In **(C)**, location of the desiccated microbial mat in a hillside of a mound at the time of collection (indicated with a white arrow). In **(D)**, one of the meltwater ponds closest to the location of the desiccated microbial mat. Active microbial mats are observed in the meltwater pond.

**Figure 2:**
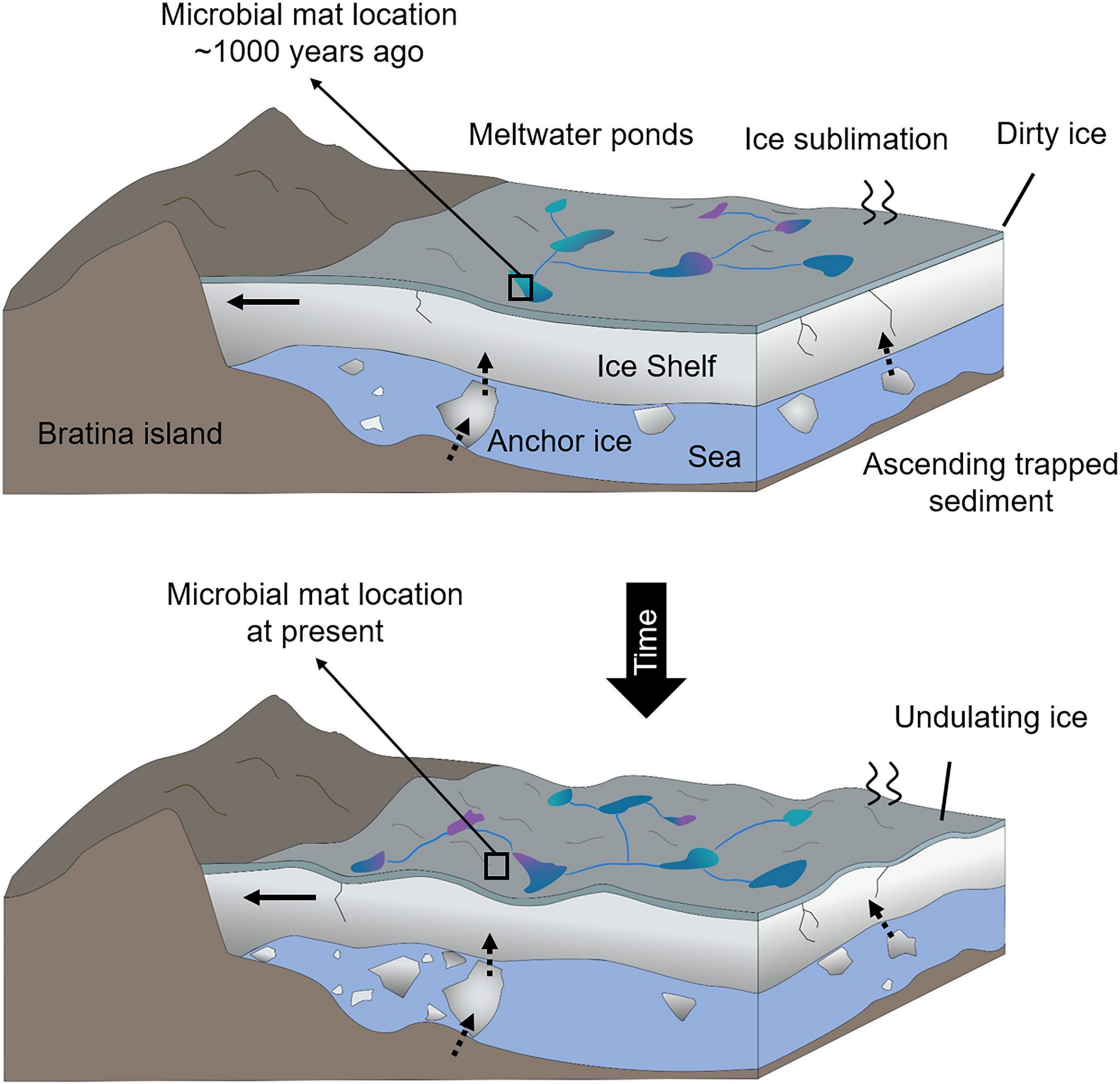
Sketch of the temporal shifts and physical processes occurring in the MIS close to the coast of Bratina Island. Horizontal black arrows in the ice shelf indicate the sense and direction of the ice compression against the coast of Bratina Island that causes the undulation of the ice. Vertical and inclined dashed arrows represent the sense and direction of the debris ascension as a result of the ice basal accretion and surface ablation. Different colors of the meltwate ponds represent a mixture of conditions (e.g., different salinities), and black boxes indicate the hypothetical location of the microbial mat ~1,000 years ago (submerged in a meltwater pond) and at present (desiccated and exposed to atmospheric conditions).

Relict microbial mats are distributed at different localities on Earth, and those that are in Antarctica offer great potential for preservation due to a combination of very low temperatures and rapid cell desiccation owing to sublimation processes (Willerslev and Cooper, 2005). An area with extensive developments of benthic microbial mats in Antarctica, some of which may remain preserved dry over years, is the McMurdo Ice Shelf (MIS). Particularly in the area close to Bratina Island, the compression of the ice shelf against land originates a deformation of the ice causing a rolling surface relief that favors the formation of a network of seasonal meltwater ponds that vary in size and salinity (Figures 1, 2; Howard-Williams et al., 1990; Vincent et al., 1993b; De Mora et al., 1994; Wait et al., 2009; Archer et al., 2014) and that are colonized by thick benthic microbial mats dominated by *Cyanobacteria* (Quesada and Vincent, 2012; Archer et al., 2015; Hawes et al., 2018; Jackson et al., 2021). As a consequence of the ice compression and plasticity, some of these microbial mats may remain distant from the water level for decades and centuries, halting their metabolisms and becoming ecological legacies of past environmental conditions in the MIS.

In a previous study, an ancient microbial mat from the McMurdo Ice Shelf (~1,000 years BP) succinctly reported an absence of cyanobacterial DNA sequences, but they were detected with a multiplex immunological assay (Blanco et al., 2017). Other ancient microbial mats (from ~2,000 to more than 26,000 years BP) located in deltas and relict shorelines of paleolakes in the nearby McMurdo Dry Valleys also reported the absence of DNA sequences from *Cyanobacteria* (Hall et al., 2001, 2002; Antibus et al., 2012b; Zaikova et al., 2019). These previous works suggest that reconstruction of past microbial communities may be incomplete when exclusively based on DNA analyses. Therefore, combining the analysis of relatively labile DNA with other more degradation-resistant biomolecules is crucial for achieving an integral reconstruction of an ancient sample. Despite some studies have combined the analysis of various molecular biomarkers in sediments and rocks (Boere et al., 2009; Sánchez-García et al., 2020, 2021), an assessment of the joint capacity of DNA, proteins and lipids to elucidate the biological composition and functioning of an ancient sample has not yet been attempted.

Thus, here we carried out an exhaustive study of biomolecular preservation on a naturally desiccated ancient microbial mat (~1,000 years BP) from the MIS near Bratina Island (Antarctica). By characterizing DNA, proteins and lipid biomarkers using DNA metabarcoding, metaproteomics, and lipid biomarker analyses, we evaluated the diagnostic capacity of each molecular method and performed a biological and metabolic paleoreconstruction of the original microbial mat microecosystem from the MIS.

## Materials and Methods

### Sampling Site and Sample Description

A naturally desiccated microbial mat was collected in the McMurdo Ice Shelf (Antarctica) close to Bratina Island (78°00′S, 165°35′E) during the austral summer season of 1996. The microbial mat was located between the meltwater ponds K081 and JAC on a hillside slope ca. 7 m high and 30–40 m away from the water level at the time of collection (Supplementary Figure 1). The microbial mat was found completely desiccated showing a laminated structure and was partially covered by a layer of sediments possibly due to the ice flexure. The sample was carefully collected aseptically in a Whirl-Pak bag, stored in the dark at room temperature, and opened only once in 2015 under sterile conditions for radiocarbon dating as well as physicochemical and mineralogical characterization (Blanco et al., 2017). Radiocarbon dating of the microbial mat provided an age of 1,070 ± 30 years BP (Blanco et al., 2017). The mineralogical analysis of the microbial mat showed a composition of phyllosilicates (montmorillonite, saponite and palygorskite) and other minerals derived from volcanic rocks (augite and anorthite). Moreover, a DNA analysis showed absence of cyanobacterial sequences (Blanco et al., 2017). Desiccation, low heterotrophic growth (see Results section) and darkness likely minimized the loss of biomolecules of this ~1,000 year-old microbial mat during ~20 years of storage.

For the present study, the microbial mat was gently disaggregated and sieved through a sterile (autoclaved) 500 μm sieve (ø 200 mm, Retsch GmbH, Haan, Germany) to remove coarse rock fragments. The fraction of the sample below 500 μm in size was homogenized and separated into eight equally weighted portions, one of which (~18 g) was used for molecular analyses and growth assays. The rest of the sample portions were earmarked for a larger study. Since the portion of the mat sample used here is part of a larger study, a pre-treatment was carried out before the analyses to homogenize procedures and enhance further sample comparison. The pre-treatment consisted of a washing step with sterile water (1:100) followed by centrifugation (21,000 × *g* for 10 min) to recover the pellet and supernatant. For the purposes of this study, and based on the relatively low concentration of DNA and proteins in the supernatant (Supplementary Figure 2) compared to the fraction in the pellet (~3-fold and ~30-fold higher, respectively. See Results section), the pellet was considered as the most representative of the sample and thus used for downstream analyses. The DNA and proteins contained in the pellet correspond to those intracellular and/or surface-bound extracellular (attached to organic or inorganic compounds, e.g., exopolymeric substances or mineral particles). Finally, a piece of the original microbial mat structure was used for fluorescence microscopy analysis.

### Fluorescence Microscopy

The structure of the mat (ca. 3 × 2 × 0.5 cm) was divided into several longitudinal sections and observed under an epifluorescence microscope (Nikon Eclipse 80i microscope, Nikon, Japan). Sample sections were hydrated with sterile Milli-Q water for 2 min, dispersed on glass slides using six replicates and visualized under the microscope using a Nikon blue filter B-2A (EX 450–490 nm, DM 505, BA 520) and a Nikon green filter G-2A (EX 510–560 nm, DM 575, BA 590) for chlorophyll and phycobiliprotein excitation.

### Growth Assays of Phototrophic and Heterotrophic Microorganisms

The viability of phototrophs in the microbial mat was tested in liquid and solid BG11 medium. About 0.5 g of sample previously homogenized was inoculated in duplicates in flasks with BG11 liquid medium. Another 0.5 g of the sample was hydrated in BG11 liquid medium and then aliquots were transferred in duplicates to BG11 medium plates with purified agar (Oxoid, Hampshire, United Kingdom). Flasks and plates were incubated for 2 months at 12°C with photosynthetically active radiation (PAR) of ~10 μmol photons m^−2^ s^−1^ under a photoperiod of 16 h, at 20°C with a constant PAR of ~7 and ~20 μmol photons m^−2^ s^−1^, and at 27°C with a constant PAR of ~3 μmol photons m^−2^ s^−1^. The different PAR intensities were achieved using a shadow mesh. Positive controls with *Chrococcidiopsis* sp. strain 029 (Billi et al., 2001) were included for the tests at 20°C and 27°C, and two negative controls with liquid and solid BG11 medium without sample were included in the experiments.

The viability of heterotrophs was tested in solid R2A and LB media. About 0.5 g of sample previously homogenized was hydrated in sterile PBS buffer (Biosolve Chimie, Dieuze, France) and then aliquots of 10-fold dilutions were transferred to R2A and LB agar media. Plates were incubated at 15°C, 20°C, and 25°C in parallel in dark conditions for 20 days. Three positive controls of *Rhodococcus* sp. JG3 (Goordial et al., 2015) and three negative controls without sample were included in the experiments.

### Genomic DNA Extraction

Genomic DNA of the microbial mat was extracted in triplicates (0.6 g each) after the pre-treatment described above (i.e., washing with water and centrifugation step to separate pellets and supernatants). Pellets were used for the extraction of intracellular and/or surface-bound extracellular DNA. Negative control of the extraction process without microbial mat material was also processed in parallel. The pellet was divided into two subsamples to increase extraction yield. Genomic DNA was extracted using the DNeasy PowerBiofilm kit (QIAGEN, Hilden, Germany) following the manufacturer’s instructions with several modifications (Supplementary Text 1) to enhance extraction of DNA from thick cells and recover sequences from *Cyanobacteria* (previously unsuccessful in Blanco et al., 2017). Quantification of the DNA was performed with a Qubit dsDNA BR Assay kit (Invitrogen, Thermo Fisher Scientific, Waltham, MA, United States), and samples were stored at −20°C until analysis. The negative control of the extraction (without sample material) showed DNA concentration below the quantification limit (10 pg·μl^−1^). Equal concentrations of the three DNA extractions of the microbial mat were combined and sent for PCR and sequencing to the Genomic Service in Madrid Science Park Foundation (Spain). In addition, the negative control of the extraction (without sample) was also sent for PCR and sequencing to the Genomic Service.

### PCR Amplification and Illumina MiSeq Sequencing

Bacterial, archaeal and eukaryotic composition in the microbial mat was analyzed by the construction of paired-end SSU rRNA gene amplicon libraries and sequenced on Illumina MiSeq platform (Illumina Inc., San Diego, CA, United States). Purified DNA was quantified by Picogreen (Invitrogen) and then 3 ng of DNA was used as input in a first PCR using a Q5 Hot Start High-Fidelity DNA Polymerase kit (New England Biolabs, Massachusetts, United States). The bacterial 16S rRNA gene was amplified using the primer pair 341-F/805-R (Herlemann et al., 2011) targeting the V3–V4 hypervariable region. The archaeal 16S rRNA gene was amplified using the primer pair Arch1F/Arch1R (Cruaud et al., 2014) targeting the V2–V3 hypervariable region. Finally, the eukaryotic 18S rRNA gene was amplified using the primer pair 563F/1132R (Hugerth et al., 2014) targeting the V4–V5 hypervariable region. These primers have been previously used to prospect bacterial, archaeal and eukaryotic diversity in environmental samples (Lezcano et al., 2019). PCR conditions for the three genes are described in Supplementary Text 1. The negative control of the extraction (without microbial mat material) showed absence of genes amplification and was therefore not sequenced.

Equimolar pools of each gene were purified by agarose gel electrophoresis (particularly the 18S rRNA gene, to remove an unspecific band at ~524 bp), and AMPure XP Beads (Beckman Coulter, Pasadena, United States). Amplicon pools were titrated by qPCR using the Kapa-SYBR FAST qPCR kit for LightCycler480 (Merck KgaA, Darmstadt, Germany) and a reference standard for quantification. Final amplicon pools were denatured before seeding on a flowcell and were sequenced using the MiSeq reagent kit v3 (Illumina Inc. San Diego, CA, United States) in a 2 × 300 pair-end sequencing run on an Illumina MiSeq sequencer (Illumina Inc.).

### DNA Metabarcoding Analysis

Raw reads obtained from Bacteria (127,694), Archaea (159,465) and Eukarya (168,114) were processed in Mothur software v.1.44.2 (Schloss et al., 2009) following the MiSeq SOP pipeline (Kozich et al., 2013). Forward and reverse reads were merged and quality-filtered. Quality-filtering criteria applied were: (i) removal of reads below 400 bp for Bacteria, 300 bp for Archaea and 500 bp for Eukarya, (ii) ambiguous nucleotides, (iii) homopolymers longer than 8 bp, and (iv) chimeras scanned with VSEARCH (Rognes et al., 2016). Final sequences were 74,746 for Bacteria, 106,255 for Archaea and 47,132 for Eukarya, and were clustered into OTUs at 97% of similarity. Sequencing depth was checked using iNEXT online (Chao et al., 2016). Taxonomic annotation was performed by comparing representative OTU sequences of Bacteria and Archaea with the RDP reference database (v.16; release 11; Cole et al., 2014), as it is extensively used for taxonomic assignment of bacterial and archaeal sequences. In addition, sequences of bacteria and archaea were also analyzed with the SILVA database (v.138; Quast et al., 2013) to check for consistency of the prokaryotic community structure. Representative OTU sequences of Eukarya were compared against the SILVA reference database (v.138), which has a comprehensive repository for eukaryotic sequences. Output OTUs assigned to “cyanobacteria/chloroplast” with the RDP database were further compared with NCBI GenBank for more precise cyanobacteria taxonomic identification. Finally, singletons and sequences that were assigned to non-bacterial, non-archaeal, or non-eukaryotic entities in their respective gene libraries were removed to avoid incorrect results and conclusions. Therefore, final data debugging resulted in the removal of 0.8% of the sequences in the bacterial library (0.76% were singletons), 33.2% in the archaeal library (32.83% were bacterial sequences) and 20.7% in the eukaryotic gene library (all were singletons).

### Protein Extraction and Analysis

The metaproteome of the microbial mat sample was extracted in triplicates (ca. 1 g each) after the pre-treatment (i.e., washing with water and centrifugation step to separate pellets and supernatants). Pellets were used for the extraction of intracellular and/or surface-bound extracellular proteins. Negative control of the extraction process without microbial mat material was also processed in parallel. The pellets were extracted with sodium dodecyl sulfate (SDS) lysis and precipitated with trichloroacetic acid (TCA) following Hultman et al. (2015) with several modifications to increase the extraction yield in this sample (Supplementary Text 1). Quantification of proteins was performed with a Qubit Protein Assay kit (Invitrogen, Thermo Fisher Scientific) and samples were stored at −80°C until analysis. The negative control of the extraction showed protein concentration below the quantification limit (1 ng·μl^−1^). One protein extraction of the microbial mat sample and the negative control were sent for sequencing to the Proteomics Unit of Complutense University of Madrid (Spain).

Total protein biomass was digested in-gel with trypsin (Supplementary Text 1), and final desalted protein digests were analyzed using a nano Easy-nLC 1,000 system (Thermo Fisher Scientific) coupled to a high-resolution Q-Exactive HF hybrid quadrupole-Orbitrap mass spectrometer (Thermo Fisher Scientific). Pre-columns and columns, as well as mobile phase conditions and data acquisitions, are detailed in Supplementary Text 1.

### Metaproteomic Analysis

Mass spectra were analyzed using Proteome Discoverer v.2.3 software (Thermo Scientific) with search engine MASCOT v.2.6.1 to identify proteins using the Swiss-Prot reference database (downloaded on 5 May 2019; The UniProt Consortium, 2019). Moreover, the Contaminant database in MASCOT was used for the identification of the most common contaminants. Search parameters were peptide mass tolerance of 10 ppm, fragment ion mass tolerance of 0.02 Da, and up to two missed trypsin cleavage sites allowed. Carbamidomethylation of cysteine was specified as fixed modification, and oxidized methionine and loss of N-terminal methyl- and acetyl- were set as variable modifications. Acceptance criteria for protein identification were performed using the Percolator algorithm with a False Discovery Rate (FDR) <1% and at least one unique peptide identified with confidence (CI >99%, *q*-value <0.01).

Proteins identified in the Contaminants database and the negative control of the extraction (without microbial mat material) were removed from the microbial mat data set for downstream analysis. The negative control was mainly comprised of eukaryotes (99%), among which 86% were annotated to the phylum *Chordata*, mostly to the genus *Homo* (56%; Supplementary Figure 3). Therefore, any protein from the phylum *Chordata* in the microbial mat sample (39 proteins) was also removed for downstream analyses to assure the removal of potential human contamination during sample extraction and analysis. In addition, a small protein of 15 amino acids was also removed from the sample data set to avoid potential erroneous taxonomic assignation (phylum *Streptophyta*, order *Fabales*). A list with the original set of proteins, indicating those that were removed and those that were kept for downstream analyses is in Supplementary Table 1. Protein relative abundances were calculated as Normalized Spectral Abundance Factor (NSAF), i.e., the ratio of peptide-spectrum count number (SC) over protein length (L), and then divided by the sum of all SC/L in the sample (Zybailov et al., 2006).

Functional assignments of proteins were performed by retrieving KEGG (Kyoto Encyclopedia of Genes and Genomes) identifiers from UniProt IDs using the identifier cross-reference tool.^1^ Then, protein KEGG IDs were used to obtain KEGG pathways map annotations. The list of KEGG pathways maps was obtained using the KEGGREST package in R (Tenenbaum and Maintainer, 2020) and we selected those related to “Metabolism,” “Genetic information processing,” “Environmental information processing,” and “Cellular processes” as relevant for this study. The relative abundance of KEGG pathways in the microbial mat sample was calculated based on the sum of SAFs of the proteins that are annotated to each KEGG category. Since a protein can be involved in different metabolic pathways and the preference for one pathway or another in the microbial mat is unknown, the same probability of this protein to belong to each KEGG pathway has been assigned.

### Geolipid Extraction, Fractionation and Analysis

About 2 g of the microbial mat was extracted after freeze-drying the pellet resulting from sample pre-treatment. Lipids of the ancient mat were extracted following the method described in Grimalt et al. (1992) with modifications. Total lipids were extracted with ultrasound sonication (3 × 15 min) using a mixture of dichloromethane (DCM) and methanol (MeOH) in a proportion of 3:1 (v/v). Two organic-solvent extractions without sample were used as negative controls. Tetracosane-D_50_, myristic acid-D_27_ and 2-hexadecanol were employed as internal standards, adding them all before the sonication. The total lipid extract (TLE) was concentrated to ca. 2 ml by rotary evaporation and then treated with activated copper overnight to remove elemental sulfur. After sulfur removal, the TLE was concentrated to 0.5 ml with an N_2_ flow and hydrolyzed overnight with 35 ml of 6% KOH:MeOH (Grimalt et al., 1992). The neutral fraction was separated from the acidic fraction by successively extracting with *n*-hexane (Hx, 3 × 30 ml), and then separated into non-polar (hydrocarbons) and polar (alcohols) fractions by eluting on an alumina column (ca. 0.5 g of Al_2_O_3_ powder in a Pasteur pipet) with Hx:DCM (9:1, v/v) and DCM:MeOH (1:1, v/v), respectively. The acidic fraction (mostly alkanoic acids) was recovered from the remaining hydrolyzed-TLE by adding HCl until pH ~ 2 and then extracting with successive additions of *n*-hexane (3 × 30 ml). Whereas the non-polar fraction did not need any further preparation for analysis (Gas Chromatography–Mass Spectrometry, GC–MS), the acidic fraction was treated prior to analysis with BF_3_ in MeOH to produce fatty acids methyl esters (FAME), and the polar fraction was treated with N,O-Bis (trimethylsilyl) trifluoroacetamide (BSTFA) to produce trimethylsilyl alcohol derivatives. All solvents (solvent grade 99.9%) were supplied by Sigma-Aldrich (Madrid, Spain).

The three lipid polarity fractions were analyzed with gas chromatography (GC)-mass spectrometry (MS) using a 6,850 GC System coupled to a 5975C VL MSD Triple-Axis detector (Agilent Technologies, Santa Clara, CA, United States) that operated in conditions described in Sánchez-García et al. (2018). Compound identification was based on retention time and mass spectra comparison with reference materials and the NIST mass spectral database. Quantification was performed with the use of external calibration curves of *n*-alkanes (C_10_–C_40_), fatty acids methyl esters (FAMEs, C_6_–C_24_) and *n*-alkanols (C_14_, C_18_, and C_22_), all supplied by Sigma-Aldrich (Madrid, Spain). The mean recovery of the internal standards was 74 ± 19%.

### Elemental and Stable-Carbon Isotopic Composition

Stable-carbon isotope composition of the organic carbon (δ^13^C) and total nitrogen (δ^15^N) was measured on the freeze-dried microbial mat (ca. 0.15 g) using isotope-ratio mass spectrometry (IRMS) following the USGS method (Révész et al., 2012). The sample was homogenized by grinding using a mortar and a pestle and then decarbonated with HCl (3 M). After equilibration for 24 h, the sample was adjusted to neutral pH with ultrapure water and dried out at 50°C until constant weight. The δ^13^C and δ^15^N ratios were determined in triplicates in a MAT 253 IRMS (Thermo Fisher Scientific) and reported in parts per mil (‰). Three certified standards were used (USGS41, IAEA-600, and USGS40) with an analytical precision of 0.1‰. The content (%) of total organic carbon (TOC) and total nitrogen (TN) was measured during stable isotope analysis using an elemental analyzer Flash HT (Thermo Fisher Scientific).

Besides, stable-carbon isotopic composition (δ^13^C) was measured on individual *n*-alkanoic acids by coupling the gas chromatograph Trace GC 1310 ultra (Thermo Fisher Scientific) to the MAT 253 IRMS. The conditions for the GC and IRMS analysis were described in Supplementary Text 1. The isotopic values of the individual *n*-alkanoic acids separated by GC were calculated using CO_2_-spikes of known isotopic composition. Reference mixtures of FAMEs (F8, Indiana University, United States) were measured to check the accuracy of the isotopic ratio. The δ^13^C ratio was calculated by correcting the FAME values for one carbon added during the BF_3_ methylation for FAME analysis (Abrajano et al., 1994).

## Results

### Morphological Characterization and Viability of Microorganisms in a ca. 1,000 Years Old Antarctic Microbial Mat

**Figure 3:**
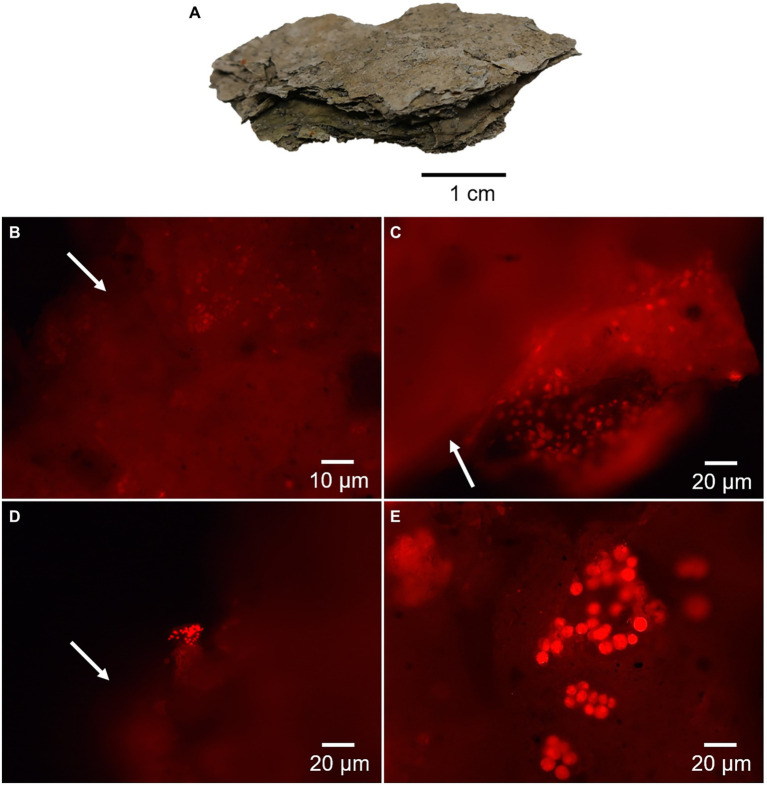
Fluorescence microscopy images of the desiccated microbial mat showing red autofluorescence of chlorophyll-bearing cells. **(A)** Shows a picture of the dried and laminated microbial mat. Micrographs in **(B,C)** show remnants of photosynthetic cells with a large amount of cellular and extracellular material scattered outside the cells (arrows). The micrograph in **(D)** shows a possible colony of *Cyanobacteria* and release of photosynthetic pigments (arrow), and in **(E)**, a colony of microalgae with apparent entire cells.

During fluorescence microscope analysis, the desiccated microbial mat showed areas with an intense red autofluorescence that revealed remnants of photosynthetic microorganisms (Figure 3A). Microscope inspection mostly showed cell damage and extracellular content scattered outside the cells (Figures 3B,C). After exhaustive sample observation, certain cells aggregates were also identified, possibly related to cyanobacteria (Figure 3D) and microalgae (Figure 3E).

A growth test to evaluate the viability of phototrophic cells in the desiccated microbial mat gave negative results under the tested growth conditions. In contrast, the assays performed in the R2A medium to evaluate the growth of heterotrophs showed 18 ± 1 CFU mg^−1^ dw at 15°C, 26 ± 6 CFU mg^−1^ dw at 20°C, and 18 ± 8 CFU mg^−1^ dw at 25°C. Fewer colonies grew under the same conditions in LB medium, specifically 0.2 ± 0.3 CFU mg^−1^ dw at 15°C, and 1.0 ± 0.9 CFU mg^−1^ dw at 20°C. Colony growth was not observed at 25°C in LB medium. Both *Chrococcidiopsis* sp. 029 and *Rhodococcus* sp. JG3 used as positive controls grew in BG11 and R2A media, respectively.

### Quantification of Biomolecules in the Desiccated Microbial Mat

**Figure 4:**
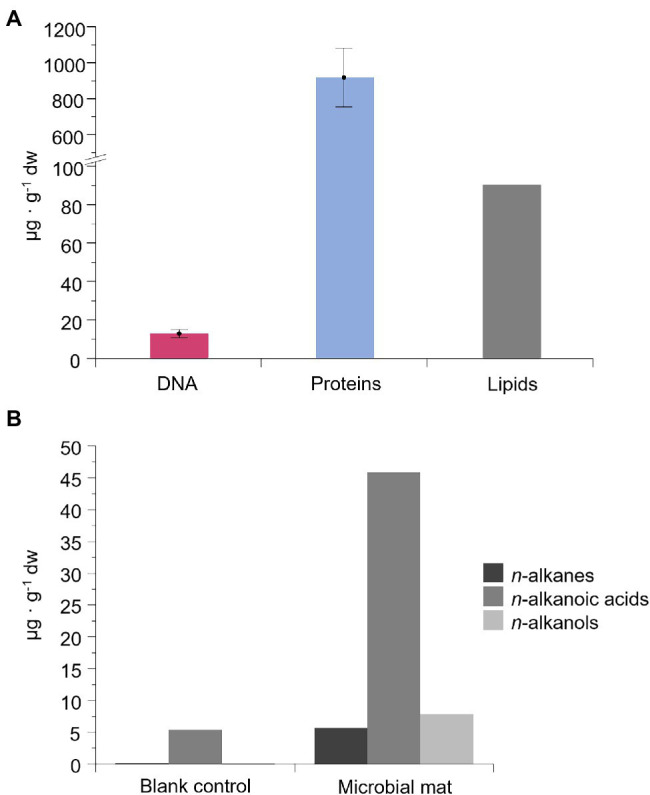
Concentration of DNA, proteins, and lipid compounds in the microbial mat (μg·g^−1^ of dry weight). In **(A)**, concentration of intracellular and surface-bound extracellular DNA and proteins, and total lipids. “Surface-bound” refers to the fraction of extracellular DNA and proteins attached to organic or inorganic compounds. Error bars are the SD of triplicates. In **(B)**, concentration of the three most abundant lipidic families: straight-chain or *normal* (*n-*) alkanes, alkanoic acids, and alkanols. Results from an organic-solvent extraction without a sample (blank control) is also represented for the lipid biomarkers.

The concentration of intracellular and/or surface-bound extracellular DNA (for more details, see the Materials and Methods section) was 13 ± 2 μg·g^−1^ dw, and that of proteins was 918 ± 163 μg·g^−1^ dw (Figure 4A). The total amount of lipids was 90 μg·g^−1^ dw (Figure 4A) and showed distinct concentrations of the non-polar, polar and acidic fractions, mostly represented by straight-chain and saturated (*normal*) alkanes, alkanoic acids, and alkanols, respectively (Figure 4B). The most abundant lipidic compounds were *n*-alkanoic acids (46 μg·g^−1^ dw), followed by *n*-alkanols (8 μg·g^−1^ dw) and *n*-alkanes (6 μg·g^−1^ dw). Thus, lipid chains containing carboxyl or hydroxyl groups were more abundant in the microbial mat than the defunctionalized hydrocarbons. The blank control showed 9-fold (*n*-alkanoic), 45-fold (*n*-alkanes) and 160-fold (*n*-alkanols) less concentration than in the microbial mat sample, thus ensuring the reliability of the signals.

### Microbial Mat Community Profiles With DNA Sequencing, Metaproteomics, and Lipid Biomarkers

**Figure 5:**
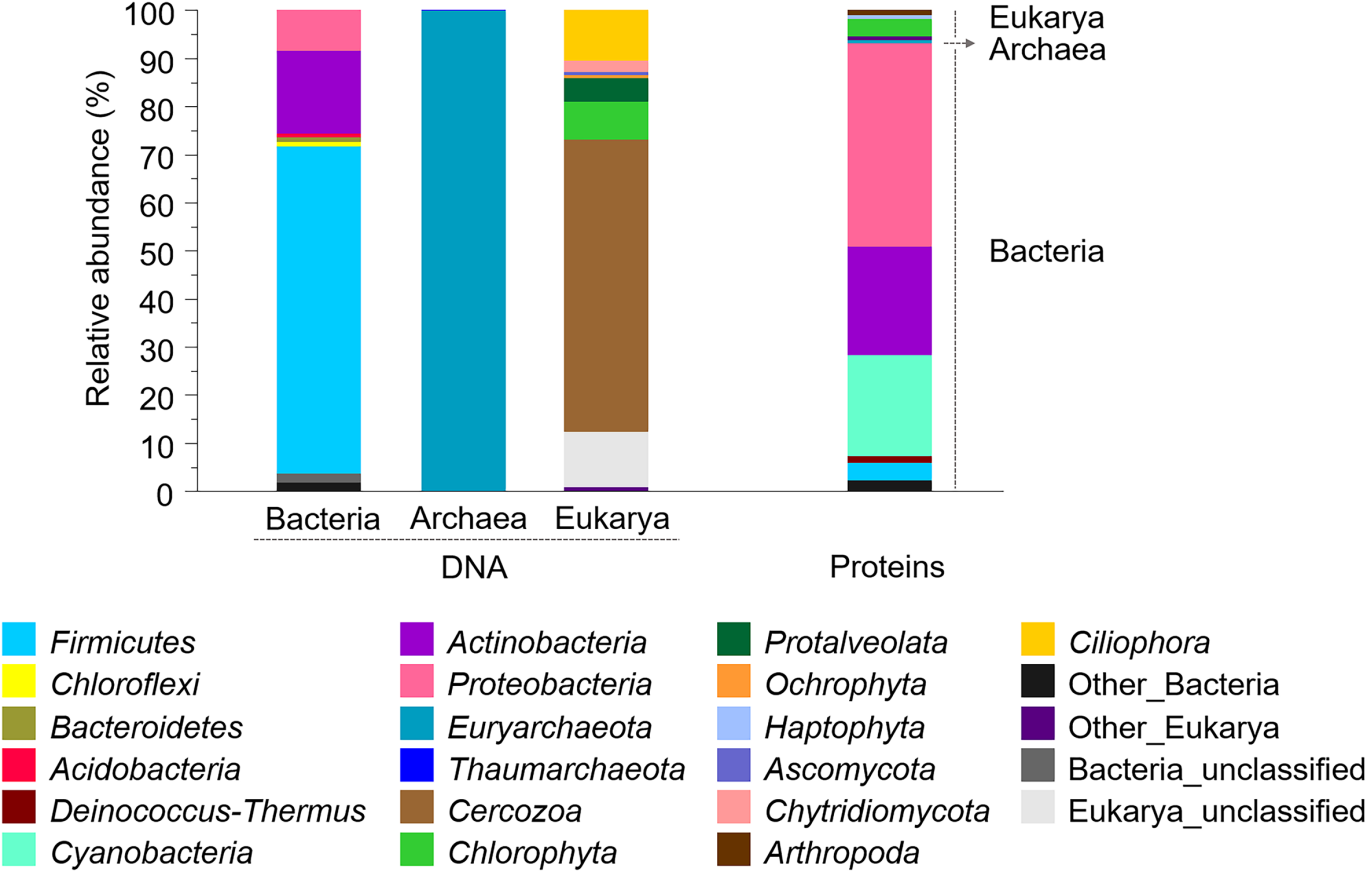
Composition and relative abundances of bacterial, archaeal, and eukaryotic phyla in the desiccated microbial mat obtained with SSU rRNA genes (left) and metaproteomics (right) analyses. For proteins, the relative abundance was calculated based on a normalized spectral abundance factor (NSAF) annotated per cent. Phyla with relative abundances below 0.5% were comprised within “Other” groups.

Three molecular approaches were applied for assessing the microbial composition of the old microbial mat. Firstly, the community structure was investigated with the DNA fraction by high-throughput sequencing of the SSU rRNA genes of Bacteria, Archaea and Eukarya. Gene amplicon sequences recovered after quality filtering were 74,175 for Bacteria, 71,029 for Archaea and 37,371 for Eukarya, and resulted in 794 bacterial operational taxonomic units (OTUs), 13 archaeal OTUs and 699 eukaryotic OTUs. The bacterial profile of the desiccated microbial mat at the phylum level showed a high relative abundance of *Firmicutes* (68%), followed by *Actinobacteria* (17%) and *Proteobacteria* (8%; Figure 5). Minor proportions of *Chloroflexi*, *Acidobacteria*, and *Bacteroidetes* were also identified (<1%). A striking observation was the relatively low abundance of *Cyanobacteria* in the desiccated microbial mat, identified at 0.009% (nine sequences, comprised within “Other Bacteria”). A second analysis of the sequences with the SILVA database also identified *Cyanobacteria* at relatively low abundance (0.015%, data not shown). The archaeal profile was dominated by *Euryarchaeota* (~100%), and the eukaryotic profile showed a large proportion of *Cercozoa* (61%), followed by *Ciliophora* (11%), unclassified eukaryotes (11%), and *Chlorophyta* (8%).

**Figure 6:**
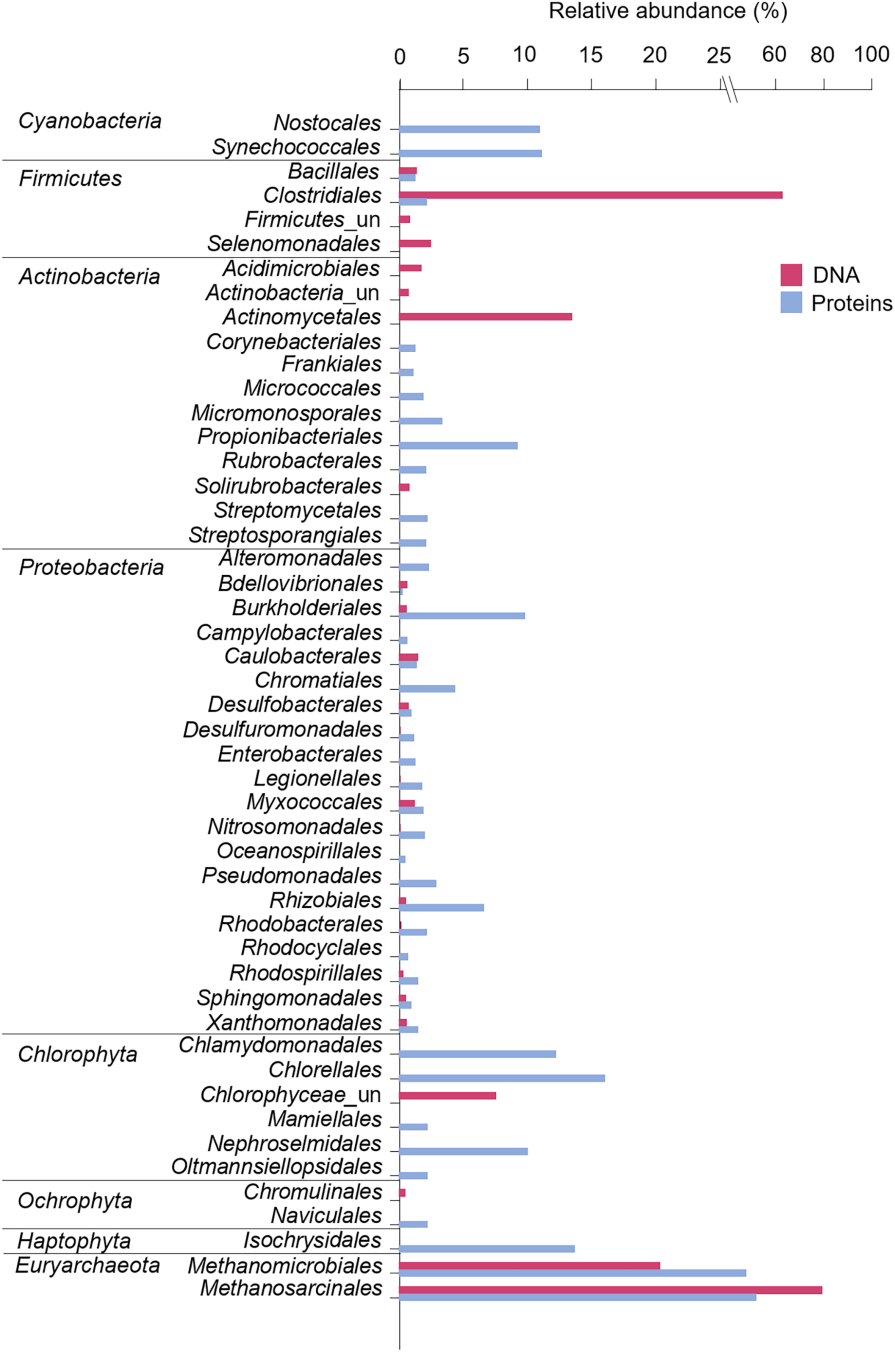
Microbial composition and relative abundance of the most abundant orders (>0.5% over the bacterial, eukaryotic or archaeal community) within the dominant phyla in the microbial mat based on DNA and protein analyses. For Eukarya, only *Chlorophyta*, *Ochrophyta*, and *Haptophyta* were represented as some of the main photoautotrophs in fresh and marine ecosystems. Relative abundances (%) of proteins were calculated based on the NSAF relative to each domain (bacteria, archaea, or eukarya). “un” means unclassified.

At the order level, the microbial mat DNA profile was dominated by *Clostridiales* within the phylum *Firmicutes*, and *Actinomycetales* within the phylum *Actinobacteria* (Figure 6). *Proteobacteria*, however, was comprised of different orders with similar relative abundances, such as *Caulobacterales*, *Myxococcales*, and *Desulfobacterales*. The phylum *Euryarchaeaota* consisted of *Methanomicrobiales* and *Methanosarcinales*. The microalgal phylum *Chlorophyta* was dominated by unclassified *Chlorophyceae*, and the phylum *Ochrophyta*, by *Chromulinales*.

As a second approach, the community structure of the desiccated microbial mat was investigated by metaproteomics analysis. The number of different proteins identified in the sample was 238, while that in the negative control was 70. After proteins of the negative control, the phylum *Chordata* and a short-length protein (explained in Materials and Methods) were subtracted, the microbial mat sample yielded 140 final non-redundant proteins with an average sequence coverage of 4.7% (Supplementary Table 1). The microbial mat metaproteome was dominated by bacteria (93%), followed by eukaryotes (6%) and archaea (1%; Figure 5). Within the bacterial profile, the phyla with the highest relative abundances were *Proteobacteria* (42%), *Actinobacteria* (23%) and *Cyanobacteria* (21%). Although relative abundances between DNA and protein profiles are not strictly comparable due to the separate analysis of bacteria, archaea and eukaryotes in the DNA (amplicons) while unified in the metaproteome, the differences in the relative abundance of *Cyanobacteria* between proteins (21%) and DNA (0.009%) were remarkable. Similarly, the relative abundance of *Firmicutes* in the metaproteome was significantly lower (4%) than that found in the bacterial 16S rRNA gene profile (68%). Within the eukaryotic and archaeal profiles, *Chlorophyta* (4%) and *Euryarchaeota* (1%) were the most abundant.

At the order level, proteins exhibited a more diverse microbial community profile than DNA (Figure 6). *Cyanobacteria* were comprised of *Nostocales* and *Synechocccales*, and the phylum *Actinobacteria* was dominated by *Propionibacterales*. The phylum *Proteobacteria* was mainly comprised of *Burkholderiales*, *Rhizobiales*, and *Chromatiales*, in addition to a wide number of orders with similar relative abundances, such as *Pseudomonadales*, *Nitrosococcales*, *Myxococcales*, and *Desulfuromonadales*. The archaea *Euryarchaeaota* consisted of *Methanomicrobiales* and *Methanosarcinales*, and microalgae were dominated by *Chorellales*, *Chlamydomonadales*, and *Nephroselmidales* (green algae), *Naviculales* (diatoms) and *Isochrysidales* (haptophytes).

**Figure 7:**
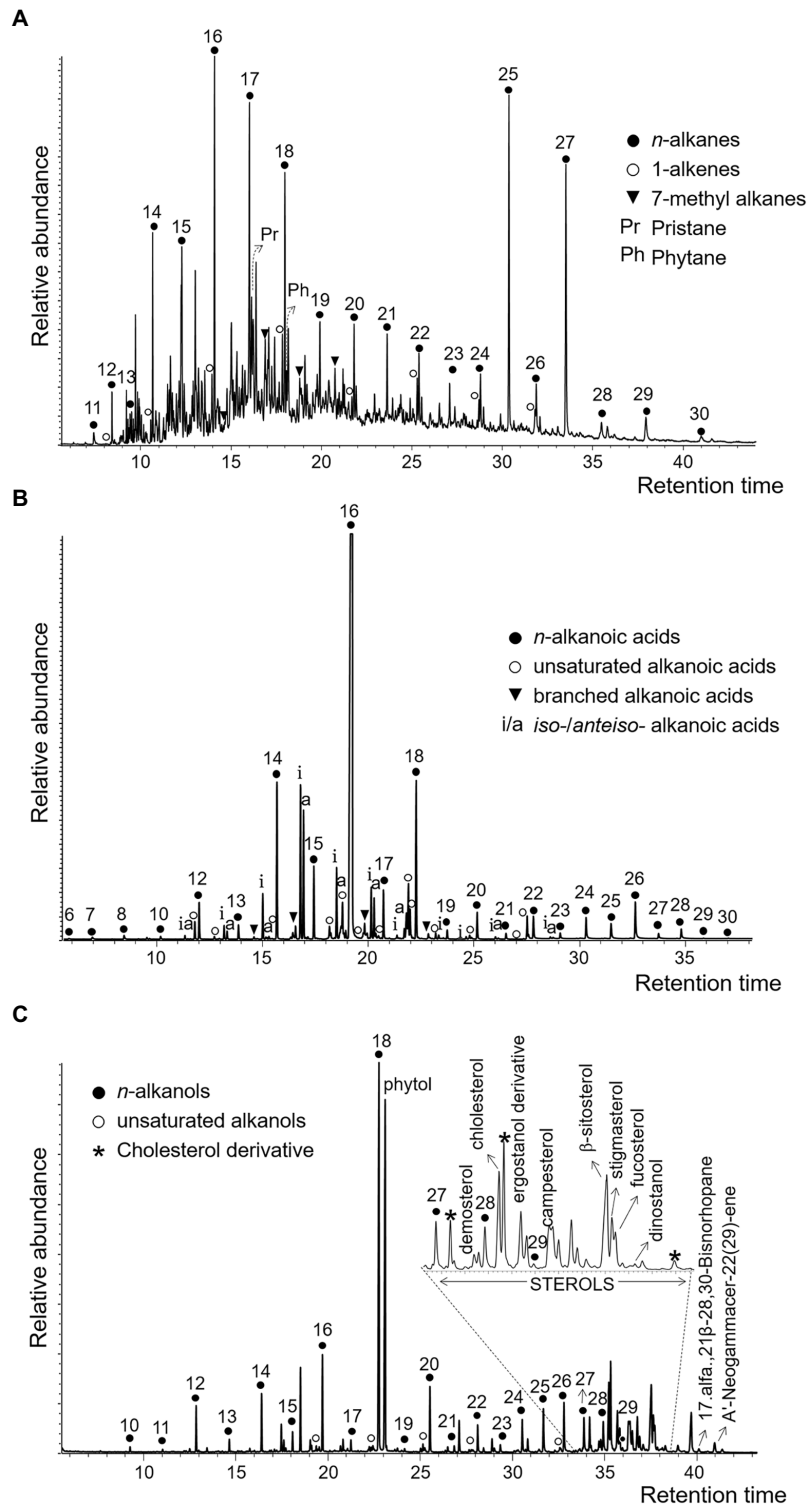
Partial ion chromatograms of the three polarity fractions of lipids in the ancient microbial mat; non-polar **(A)**, acidic **(B)**, and polar **(C)**. Hydrocarbons in **(A)** were measured in the *m/z* = 57 ion, alkanoic acids in **(B)**, in the *m/z* = 74 ion, and alcohols in **(C)**, in the *m/z* = 75 ion. In the three panels, numbers over the peaks indicate carbon chain lengths of the straight-chain or *normal* series (i.e., *n*-alkanes, *n*-alkanoic acids, and *n*-alkanols). In **(B)**, *iso*-/*anteiso*- pairs (marked as i/a over the peaks) refer to branched alkanoic acids with a methyl group in positions N-1 or N-2, respectively.

As a third approach, we analyzed the lipid biomarkers in the desiccated microbial mat. The *n*-alkanes series (non-polar fraction) showed a bimodal molecular distribution dominated by high molecular weight alkanes (HMW), mostly *n*-C_25_ and *n*-C_27_ and, to a lesser extent, low molecular weight alkanes (LMW), mostly *n*-C_16_, *n*-C_17_, and *n*-C_18_ (Figure 7A; Supplementary Figure 4). Other HMW alkanes such as *n*-C_23_ and *n*-C_29_ were also detected at lower relative abundances. A group of mid-chain monomethyl alkanes ranging from C_16_ to C_19_ was also identified, in addition to the isoprenoidal hydrocarbons pristane and phytane. In contrast to the *n*-alkanes, the molecular distribution of both the *n*-alkanoic acids (acidic fraction) and *n*-alkanols (polar fraction) showed a clear predominance of LMW over HMW compounds, as well as an even-over-odd carbon number preference. The *n*-alkanoic acids were the most abundant compounds, with the C_16:0_ acid dominating the series, followed by C_14:0_ and C_18:0_ (Figure 7B; Supplementary Figure 4). The HMW *n*-alkanoic acids showed a maximum at C_26:0_. Other alkanoic acids identified were the *iso*-/*anteiso*- pairs from C_15:0_ to C_18:0_, and the monounsaturated alkanoic acids from C_16_ to C_22_, including the monounsaturated 16:1(ω7) and 18:1(ω9). In the polar fraction, the most abundant compounds were *n*-octadecanol and phytol (Figure 7C; Supplementary Figure 4). The rest of *n*-alkanols were up to three orders of magnitude less abundant than C_18_, with peaks at C_16_ and C_20_ within the LMW moieties, and at C_26_ among those of HMW. Finally, a set of sterols indicative of eukaryotic (e.g., dinosterol, demosterol, cholesterol, ergostanol, campesterol, β-sitosterol, stigmasterol or fucosterol) and prokaryotic (the hopanoids 17α(H),21β-28,30-Bisnorhopane and hop-22(29)-ene (diploptene)) sources were also identified (Figure 7C).

### Inference of Metabolic Pathways in the Desiccated Microbial Mat

**Figure 8:**
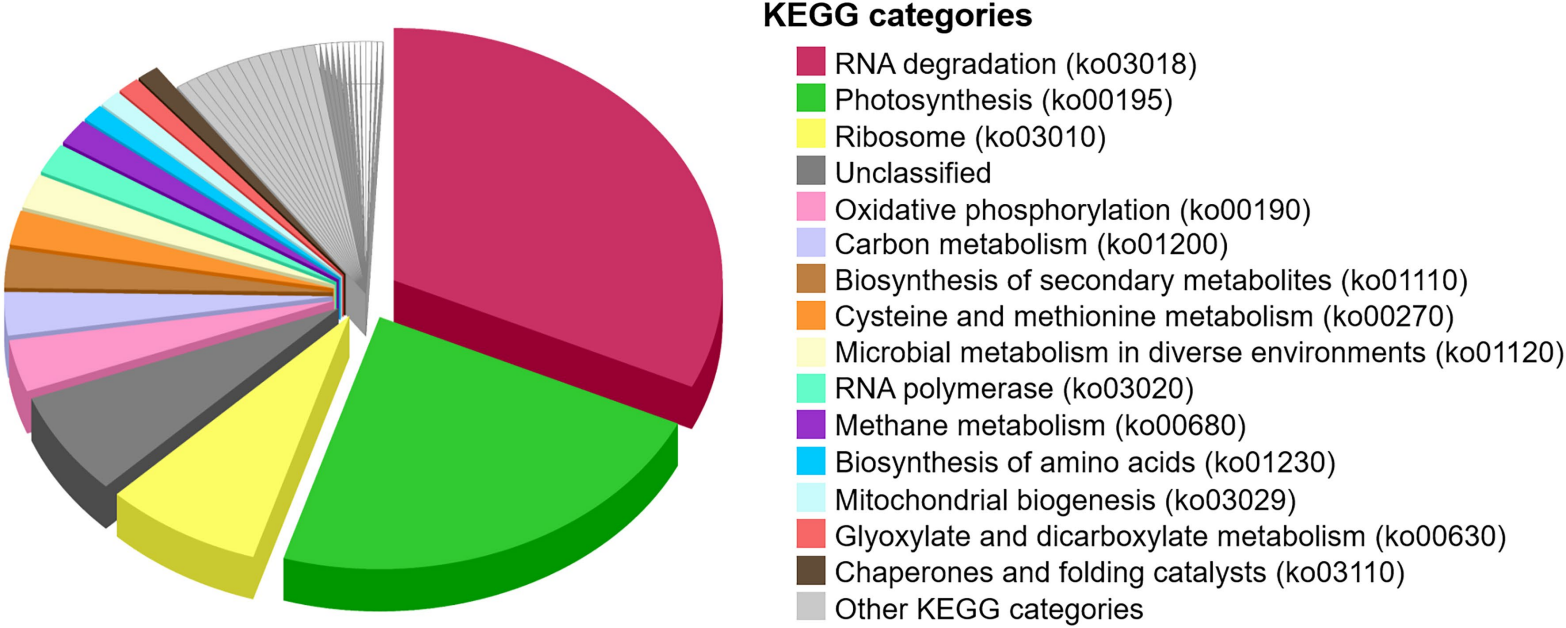
Proportion of metabolic pathways in the desiccated microbial mat based on the functional assignment of the metaproteome to KEGG pathway maps. The relative abundance of KEGG categories was calculated based on the sum of SAFs of the proteins that are annotated in each KEGG category. “Unclassified” are proteins unable to cross-reference with the KEGG database. “Other KEGG categories” are shown in Supplementary Figure 6.

Functional assignments of the metaproteome were performed to define essential protein functions and general metabolic pathways that operated in the microbial mat before desiccation ~1,000 years ago (Figure 8). The most abundant proteins were 60 kDa chaperonins (Supplementary Table 1), implicated in assisting protein folding and potentially involved in RNA degradation (32%). The following most abundant proteins were chlorophyll *a* apoproteins from photosystem I, and reaction centre proteins from photosystems I and II, all involved in photosynthesis (23%). Ribosomal proteins (30S and 50S), involved in translation, were also relatively abundant in the microbial mat (8%). Apart from other general metabolisms in the cells (e.g., oxidative phosphorylation (3%), carbon metabolism (3%), and biosynthesis of amino acids (1%)), the detection of methyl-coenzyme M reductase and F420-dependent methylenetetrahydromethanopterin dehydrogenase indicated the synthesis of methane in the microbial mat (2%). The identification of the ammonia monooxygenase also revealed nitrification processes in the microbial mat (0.5%, within “Other KEGG categories,” Supplementary Table 1).

Besides proteins, the bulk and compound-specific isotopic composition of the *n*-alkanoic acids were analyzed to assess the carbon fixation pathways that prevailed in the microbial mat. Bulk carbon and nitrogen stable isotope ratios of the biomass were −7.82 ± 0.04‰ (δ^13^C) and 4.12 ± 0.03‰ (δ^15^N). For the *n*-alkanoic acids from C_14:0_ to C_28:0_, the compound-specific values of the stable carbon ratio (δ^13^C) ranged from −6‰ to −18‰, and showed a general enrichment of ^13^C with the carbon number, except for C_16:0_ (Supplementary Figure 5). The content of TOC in the microbial mat was 3.5 ± 0.2%, and that of TN was 0.36 ± 0.03%.

## Discussion

### Long-Term Dryness Likely Dwindled Microbial Mat Viability

After organisms decay, environmental conditions (e.g., temperature, radiation, and humidity) and processes (e.g., deposition, transport, decomposition, and scavenging) can cause selective preservation of biological remains resulting in biases that affect the diversity and reconstruction of the biological community structure (Kidwell and Behrensmeyer, 1988). Given these difficulties, in this study we used an integrative approach combining analysis of different types of biomolecules (DNA, proteins and lipid biomarkers) to reliably reconstruct the taxonomic composition and metabolism of an ancient microbial mat of ~1,000 years BP from the MIS before desiccation.

According to radiocarbon analysis, the average age of the desiccated microbial mat was 1,070 ± 30 years BP (Blanco et al., 2017), although a possible reservoir effect causing an overestimation of the sample age cannot be ruled out (Hall and Denton, 2000; Hendy and Hall, 2006). Regardless of the precise microbial mat age, we hypothesize that the microbial mat was rapidly desiccated (due to the strong sublimation processes in the MIS) hundreds of years ago when the ice shelf topography changed and left the microbial mat isolated on the side of a mound away from a meltwater pond (Figure 2). Rapid cell desiccation is one of the most relevant mechanisms that allow cells to tolerate low temperatures and then resume photosynthesis and respiration when conditions are favorable (Potts, 1994; Billi and Potts, 2002). However, the negative results of the growth test in the BG11 medium with the Bratina microbial mat indicated the death or inability of photoautotrophs for growing under the tested conditions. These observations agree with earlier studies showing that cyanobacteria can survive decades of anhydrobiosis (Lipman, 1941; Hawes et al., 1992) but do not survive for longer periods (Antibus et al., 2012a). During cell desiccation, the imbalance between light-harvesting and respiration produces high-energy intermediates that give rise to reactive oxygen species (ROS; Singh, 2018; Oliver et al., 2020). Therefore, the accumulation of ROS and the incidence of intense UV radiation in this area during the microbial mat desiccation may have caused the permanent loss of photosynthetic activity.

In contrast to photoautotrophs, sparse colonies of heterotrophs grew on R2A medium (maximum of 26 ± 6 CFU mg^−1^ dw at 20°C), indicating the viability of a few heterotrophic microorganisms in the desiccated microbial mat. This number of viable heterotrophs represent ~1,000-fold fewer colonies than those observed in modern microbial mats (~8 years BP) from the nearby McMurdo Dry Valleys, and resembles more to those that dated from ~11,000 to ~26,500 years BP (Antibus et al., 2012a). Therefore, the limited viability of heterotrophs, the extremely short period of liquid water, the location of the sample at the time of collection, and the strong sublimation processes in the MIS, suggests that any rehydration event that might have occurred on the microbial mat was probably insufficient for a significant and long-term reactivation of the microbial metabolism.

### Cold, Dryness, and Salts in the MIS Likely Favoured Biomolecule Preservation

The extraction of DNA, proteins, and lipids in the desiccated microbial mat (Figure 4A) indicated substantial preservation of the three types of biomolecules. The low temperature, rapid cell desiccation, and the protective role of salts and mineral/organic particles in the MIS may have contributed to the preservation of the biomolecules. In fact, low temperatures and rapid cell desiccation have been reported to diminish *post-mortem* DNA damage (Willerslev and Cooper, 2005), characterized by spontaneous chemical modification, interstrand cross-links and base removal (Lindahl, 1993; Potts, 1994; Hansen et al., 2006). Therefore, the detection and amplification of DNA from the Bratina microbial mat suggested an influence of the cold, dry and salty environment on the MIS for slowing down molecular damage. Similarly, the low temperature and mineral binding may have also contributed to the preservation of proteins and lipids, as stated in other studies (Röling et al., 2015; Demarchi et al., 2016). Protein diagenesis through chemical reactions (e.g., peptide bond hydrolysis and amino acid racemization) and molecular breakdown increase with temperature (Demarchi et al., 2016), as does the defunctionalzation of biolipids to hydrocarbon skeletons (Brocks and Summons, 2003). Moreover, the relatively higher concentration of *n*-alkanoic acids (with carboxylic groups) over *n*-alkanes (without functional groups; Figure 4B) suggested considerable preservation of cell membrane constituents in the microbial mat for years.

### DNA, Proteins and Lipid Biomarkers Provide Distinct Taxonomic Specificity and Temporal Resolution of the Biological Remains

The microbial community structure obtained with the DNA analysis showed the detection of only nine sequences of *Cyanobacteria* (0.009% of the whole bacterial community). This low detection is striking considering the general abundance of *Cyanobacteria* in modern and fresh microbial mats from the MIS (Vincent et al., 1993b; De los Rios et al., 2004; Quesada and Vincent, 2012; Jackson et al., 2021). The relatively large cell size of photoautotrophs compared to heterotrophic bacteria may contribute to overestimate the abundance of cyanobacteria in microbial mats and thus cause unexpectedly lower detection of cyanobacterial 16S rRNA gene sequences compared to heterotrophic bacteria (Sørensen et al., 2005). However, the number of cyanobacterial sequences in the Bratina mat was extremely low (only nine) and the bacterial profile was consistent with DNA profiles from other ancient microbial mats in the McMurdo Dry Valleys (Antibus et al., 2012b; Zaikova et al., 2019), thus also suggesting alternative explanations. The successful amplification of *Cyanobacteria* in other Antarctic microbial communities with the same primers used here (Lezcano et al., 2019) rules out the possibility of an inappropriate choice of primers. Therefore, the low detection of cyanobacterial sequences rather points to a relatively lower preservation of cyanobacterial DNA remnants compared to other taxa in the Bratina microbial mat. For instance, *Firmicutes* (68%), and particularly the endospore-forming *Clostridiales* (46,773 sequences, comprising 63% of the total bacterial composition, Figure 6), dominated the DNA profile of the old microbial mat. The dominance of *Clostridiales* was also observed in the other ancient microbial mats from the McMurdo Dry Valleys (Antibus et al., 2012b). Therefore, a selective preservation of DNA from the most resistant cells (e.g., spores) appeared to occur over time, preventing the detection of the microbial mat original diversity. Indeed, the DNA of clostridial endospores is protected against damage by the binding of small, acid-soluble proteins (SASPs) of the α/β-type that limit DNA chemical and enzymatic reactivity, as well as the effects of the UV photochemistry (Setlow, 1995; Lee et al., 2008). Thus, the lower preservation of cyanobacterial DNA remnants together with preferential PCR amplification of the undamaged DNA of endospore-forming bacteria (Pääbo, 1989) likely resulted in a partial reconstruction of the original microbial mat community composition.

In contrast to the DNA analysis, metaproteomics detected *Cyanobacteria* in relatively higher abundance (21%) in the desiccated microbial mat, and was dominated by *Synechococcales* and *Nostocales* (Figure 6). The absence of *Nostocales* (which can form akinetes) in the DNA microbial profile suggested that akinetes were less resistant than *Firmicutes* endospores under the environmental conditions in the MIS, thus supporting the previous hypothesis that long-term desiccation of *Nostocales* caused DNA damage that prevented PCR amplification (Shirkey, 2003).

**Figure 9:**
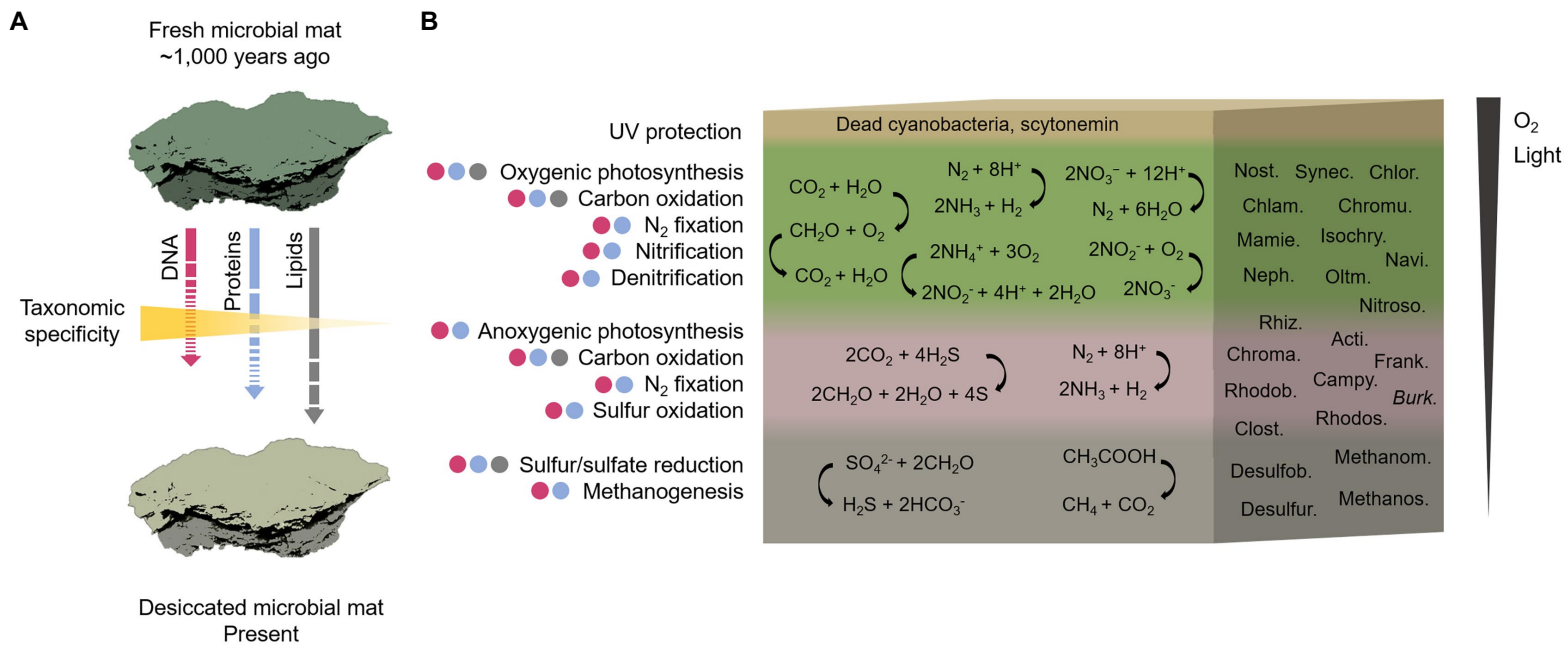
Reconstructive sketch of the biological composition and metabolisms that operated in the ancient microbial mat on the MIS before desiccation. In **(A)**, the temporal shift of the microbial mat from the hypothetical scenario of a fresh and photosynthetically active microbial mat ~1,000 years ago to the present desiccated state. The length and continuity/discontinuity of the arrows indicate an estimate of the degree of preservation of the three biomolecules studied (lipids > proteins > DNA) with an inverse gradient of taxonomic specificity. In **(B)**, reconstruction of the biological composition and metabolisms across a representative cross-section of the Bratina microbial mat. The cross-section shows a vertical stratification based on previous microbial mats descriptions (Vincent et al., 1993b; De los Rios et al., 2004; Prieto-Barajas et al., 2018). The presence of putative metabolic functions in each layer is the result of an integrative approach based on the identification of microbial taxa detected by DNA (purple circles), proteins (blue circles) and lipid biomarkers (grey circles) analyses. The metabolisms may not be exclusively associated with a certain layer. A global reaction for each metabolism is represented as an example. The most abundant taxa in the microbial mat determining the metabolisms are: *Nostocales* (Nost.), *Synechococcales* (Synec.), *Chlorellales* (Chlor.), *Chlamydomonadales* (Chlam.), *Chromulinales* (Chromu.), *Mamiellales* (Mamie.), *Isochrysidales* (Isochry.), *Naviculales* (Navi.), *Nephroselmidales* (Neph.), *Oltmannsiellopsidales* (Oltm.), *Nitrosomonadales* (Nitroso.), *Rhizobiales* (Rhiz.), *Actinomycetales* (Acti.), *Chromatiales* (Chroma.), *Frankiales* (Frank.), *Rhodobacterales* (Rhodob), *Campylobacterales* (Campy.), *Burkholderiales* (Burk.), *Rhodospirillales* (Rhodos.), *Clostridiales* (Clost.), *Desulfobacterales* (Desulfob.), *Desulfuromonadales* (Desulfur), *Methanomicrobiales* (Methanom.), and *Methanosarcinales* (Methanos.) For detailed information regarding the metabolisms inferred by the microbial taxa identity, see Supplementary Text 3.

Together with the identification of *Cyanobacteria*, the metaproteomics also detected *Proteobacteria* and *Actinobacteria* at relatively higher abundances than *Firmicutes*. Since this is the first report on metaproteomics analysis on a microbial mat from the MIS and surroundings, we cannot make comparisons with other microbial mat proteomes. In an attempt to overcome these difficulties, the metaproteomic profile was compared with those of DNA from other microbial mats from the MIS and adjacent areas. Such comparison revealed that the metaproteome of the ancient Bratina microbial mat allowed the reconstruction of a microbial community structure that resembles that of modern microbial mats from the MIS (Archer et al., 2015; Jackson et al., 2021) and the Dry Valleys (Antibus et al., 2012b; Zaikova et al., 2019). This modern-like composition suggests that proteins (despite their lower taxonomic specificity compared to DNA, detailed in Supplementary Text 2) may have retained information from the original microbial composition, both because they represent a higher proportion of cell biomass than DNA and because proteins may have had lower taphonomic alteration compared to nucleic acids occurring since microbial mat desiccation. The higher preservation of proteins compared to DNA in the MIS environment (Figure 9A) is supported by previous studies that determined higher limits for the persistence of proteins (several millions of years; Rybczynski et al., 2013; Demarchi et al., 2016) than for DNA (up to 1 million years in cold conditions; Willerslev and Cooper, 2005).

Compared to DNA and proteins, lipid biomarkers have lower taxonomic specificity (i.e., able to distinguish only between general organismic sources) but higher preservation potential (Brocks and Summons, 2003; Figure 9A). In the desiccated microbial mat, the joint detection of several lipid biomarkers often associated with cyanobacteria (e.g., alkane *n*-C_17_, 7-methyl alkanes from C_16_ to C_19_ (Jahnke et al., 2004) and 16:1(ω7) and 18:1(ω9) alkanoic acids (Grimalt et al., 1992; Pagès et al., 2015)) suggest the presence of remnants of these microorganisms. The occurrence of diatoms was also suggested by the detection of dinosterol (Volkman, 2003). Moreover, other phytosterols (campesterol, β-sitosterol and stigmasterol) supported the presence of micro-/macro-algae (Martin-Creuzburg and Merkel, 2016; Taipale et al., 2016; Pereira et al., 2017; Randhir et al., 2020), and/or potentially also remnants of aquatic macrophytes (Maciel et al., 2016; Serviere-Zaragoza et al., 2021), peat vegetation including mosses (Nott et al., 2000; Pancost et al., 2002), and/or higher plants (Eglinton and Hamilton, 1967; Hedges and Prahl, 1993) according to the detection of straight-chain alkanes of HMW (*n*-C_23_, *n*-C_29_, and mostly *n*-C_25_ and *n*-C_27_; Figure 7A; Supplementary Figure 4). The association of these lipid compounds (LMW alkanes, monounsaturated alkanoic acids, and phytosterols) with cyanobacteria and microalgae sources is consistent with the DNA and/or protein microbial community profiles. However, the synthesis of alkanes of HMW by microalgae is limited (Metzger et al., 1991; Lichtfouse et al., 1994), thus their detection in the desiccated microbial mat suggest a potential contribution of mosses (mainly *n*-C_25_ and the less abundant *n*-C_23_) and/or vascular plants remnants (mainly *n*-C_27_ and the less abundant *n*-C_29_). Similarly, the identification of alkanes of HMW in microbial mats from other locations was also attributed to remnants of higher plants (Rontani and Volkman, 2005; Pagès et al., 2015).

The detection of potential mosses, and/or aquatic/land plant-derived *n*-alkanes of HMW in the desiccated microbial mat may be interpreted as a result of either aerial transport of plant material currently covering distant Antarctic lands and/or formerly covering the surrounding MIS lands masses upon a warmer climate. At present, mosses are widely distributed in moist habitats in Antarctica (Velázquez et al., 2013; Singh et al., 2018), and vascular plants are located in Maritime Antarctica (Cabrerizo et al., 2016; Carrizo et al., 2019). Even on the MIS, mosses could have grown nearby the microbial mat when the mat was located at the water pond level (Figure 2). However, the striking relative abundance of HMW alkanes (mostly *n*-C_25_ and *n*-C_27_; Figure 7A; Supplementary Figure 4) despite the absence of homologous compounds of comparable intensity in the *n*-alkanoic acids (Figure 7B; Supplementary Figure 4) and *n*-alkanols (Figure 7C; Supplementary Figure 4) series, results difficult to explain solely by an allochthonous input of fresh higher plant remnants. While the reason for the high relative abundance of *n*-C_25_ and *n*-C_27_ remains inconclusive, their provenance could be associated with contributions from ancient plant biomass. Within the three lipidic families, alkanes are the most resistant to decay over time (Brocks and Summons, 2003), being able to survive up to billions of years (Brocks et al., 2003), which makes them useful tracers of the oldest biosignatures in an ancient sample. In the McMurdo Dry Valleys, a previous study identified long-chain *n*-alkanes in soil samples (Matsumoto et al., 1990) and was argued to be associated with the erosion of sedimentary material containing vascular plants from pre- and inter-glaciation periods in Antarctica (Miocene–Pliocene). Although the molecular distribution pattern observed by Matsumoto et al. (1990) (unimodal, with a maximum at *n*-C_25_) was not identical to that observed in the desiccated microbial mat from Bratina (bimodal distribution with high relative abundance at *n*-C_25_ and *n*-C_27_), the presence and relative abundance of the odd HMW alkanes in the relict microbial mat may similarly stem from aquatic macrophytes, mosses or even land vascular plants that inhabited the MIS adjacent lands in warmer periods, for instance, during the Mid-Miocene (Lewis et al., 2008; Warny et al., 2009; Feakins et al., 2012) or early Eocene (Pross et al., 2012). In this scenario, the highly resistant plant-derived hydrocarbons would have been preserved in the marine sediments over time (as reported from the preservation of terrestrial palynomorphs in the Ross sea sediments in Warny et al., 2009 and Feakins et al., 2012) and could have been incorporated on the MIS surface (and in turn, in the microbial mat) by basal accretion of seabed sediments and ice surface ablation (Figure 2).

Alternatively to the presence of mosses and vascular plants remnants, heterotrophic microorganisms including fungi have been linked to the production of long-chain *n*-alkanes (Oró et al., 1966; Li et al., 2018) and *n*-alkanoic acids (Chen et al., 2019), and may have contributed to the provenance of HMW alkanes in the ancient microbial mat.

### An Integrative Approach Combining DNA, Protein and Lipid Information Provided a Complete Metabolic and Taxonomic Reconstruction of the Desiccated Microbial Mat

Our results showed that the distinct preservation capacity and taxonomic resolution of DNA, proteins and lipid biomarkers (Figure 9A), together with the inherent methodological bias of each analysis (i.e., DNA metabarcoding, metaproteomics and lipid biomarkers analysis), may considerably impact the biological and functional reconstruction of an ancient sample. Therefore, the combination of the diagnosis and preservation potential of the three biomolecules may improve the taxonomic and metabolic reconstruction of the ancient microbial mat from the MIS.

The reconstruction of the community structure of the Bratina microbial mat inferred from the three biomolecule analyses revealed a network of interconnected metabolisms carried out by a diverse microbiome (Supplementary Text 3). Considering oxygen and light requirements of each metabolism, we reconstructed the ecological functioning of the ancient microbial mat along a tentative mat cross-section (Figure 9B) according to previously described fresh microbial mats in Antarctica and other regions (Vincent et al., 1993b; De los Rios et al., 2004; Prieto-Barajas et al., 2018).

Primary productivity was distributed across the vertical profile, according to the evidence for oxygenic and anoxygenic photosynthesis, as well as chemoautotrophy. A large number of protein remnants in the microbial mat were involved in oxygenic photosynthesis (Figure 8; Supplementary Table 1). The detection of photosynthetic proteins together with the abundance of cyanobacteria, green algae, diatoms and dinoflagellates in the Bratina microbial mat, and the identification of cyanobacteria and microalgae in other fresh microbial mats from the MIS (Vincent et al., 1993a; De los Rios et al., 2004; Jungblut et al., 2005, 2009), suggests the oxygenic photosynthesis as an essential pathway sustaining primary production in the MIS. Such photoautotrophs may have had limited access to CO_2_ to make photosynthesis, which may explain the relatively enriched δ^13^C values both measured on the total biomass (−8‰) and especially in the alkanoic acid C_16:0_ (−6‰) compared to typical values of cyanobacteria and microalgae-rich biomass (from ca. −18‰ to −30‰; Hayes, 2001). In environments with limited diffusion of CO_2_, as may occur in microbial mats due to the slime coating of polysaccharides (Schidlowski et al., 1984), the isotopic discrimination against ^13^C during carbon fixation is lower in comparison to environments with abundant carbon supply for photosynthesis, thus causing relatively more enriched δ^13^C values (Des Marais and Canfield, 1994). In addition, an excess of DIC demand relative to the local DIC supply during carbon assimilation may also cause limitation of CO_2_ which, in turn, could result in low ^13^C isotopic discrimination. Therefore, the high δ^13^C values of the biomass and C_16:0_ alkanoic acid in the Bratina microbial mat may be the result of a photosynthesis operated under limited CO_2_ concentration. This CO_2_-diffusion limitation has also been described in other benthic cyanobacterial mats from the McMurdo Dry Valley lakes (Lawson et al., 2004). Alternatively or complementarily, other carbon fixation pathways besides the Calvin cycle used by cyanobacteria and microalgae (δ^13^C of ca. −18‰ in phytoplankton; Hayes, 2001), such as the 3-hydroxypropionate bicycle (δ^13^C from −4‰ to −15‰; Van der Meer et al., 2000) or the reverse tricarboxylic acid pathway (δ^13^C from −12‰ to −21‰; Preuß et al., 1989), could have also contributed to the relatively high δ^13^C values in the Bratina microbial mat.

Other metabolisms inferred from DNA, proteins and lipid biomarker analyses to have occurred throughout the ancient microbial mat were carbon oxidation, nitrogen fixation, nitrification, denitrification, sulfur reduction and oxidation, and methanogenesis (Figure 9B; Supplementary Text 3). Organic matter remineralization was supported by a large number of heterotrophs in the mat (e.g., most bacteria, ciliates, and fungi; Figure 5). In addition, the general enrichment of ^13^C with increasing carbon number in the alkanoic acids (from −18‰ to −6‰) may reflect heterotrophic metabolism in the microbial mat, as similarly reported in sediments and microbial mats from Antarctic lakes (Chen et al., 2019). The carbon isotopic fractionation of *n*-alkanoic acids during biosynthesis generally causes a depletion in ^13^C with increasing chain length (Monson and Hayes, 1982). Instead, the opposite trend observed in the ancient microbial mat from the MIS suggests that the relatively enriched HMW *n*-alkanoic acids may have resulted from a heterotrophic metabolism (Chen et al., 2019).

The identification of *Nostocales* and *Clostridiales* also suggested nitrogen fixation in the microbial mat, reinforcing the previous hypothesis on the importance of nitrogen input in the N-limited MIS ecosystem (Fernández-Valiente et al., 2001). Moreover, nitrification (e.g., *Nitrospira* and *Nitrosomonas*) and denitrification (e.g., *Tetrasphaera*) likely played an important role in a sustainable nitrogen budget in the microbial mat. Anoxygenic photosynthesis appeared also relevant in the dried microbial mat, according to the detection of purple sulfur (e.g., the sulfur-oxidizing bacteria *Chromatiales*) and nonsulfur (e.g., *Rhodobacterales*, particularly *Dinoroseobacter*) bacteria. The identification of the *n*-alkanoic acids i/a-C_15_, i/a-C_16_, i/a-C_17_ and i/a-C_18_ (Figure 7B), together with the identification of *Desulfobacterales* and *Desulfuromonadales* with DNA or proteins, also suggested sulfur and sulfate-reducing processes (Taylor and Parkes, 1983; Kaneda, 1991) in the microbial mat. The presence of bacterial sulfate reducers based on the detection of i/a-C_15_ and i/a-C_17_ acids and other more specific biomarkers (i-C_17:1_(ω8) and 10Me-C_16:0_) was also reported in active microbial mats from the MIS (Jungblut et al., 2009). Moreover, the detection of *Methanomicrobiales* and *Methanosarcinales*, together with the identification of enzymes involved in the synthesis of methane (e.g., methyl-coenzyme M reductase; Supplementary Table 1), suggested the production of methane in the bottom layers of the Bratina mat, thus confirming previous evidence for methanogenesis in sediments from the MIS (Mountfort et al., 2003).

## Conclusion

Environmental conditions and degradation processes after the death of organisms can cause selective preservation of biological remains thus limiting reliable paleobiological reconstructions of ancient samples. Analytical biases also pose a challenge for reconstructing microbial community structures with confidence. The integrative approach we used here by combining 16S and 18S rRNA gene sequencing, metaproteomics and lipid biomarker analyses in the ancient microbial mat from the MIS overcome the limitations in the taxonomic specificity, preservation capacity and methodological analysis of each biomolecule type, thus achieving a more comprehensive taxonomic and metabolic reconstruction of the sample than that used with biomolecules types separately. In particular, our results in the microbial mat dated to ~1,000 years BP showed selective preservation of DNA remnants from the most resistant taxa (i.e., spore-formers). In contrast, metaproteomics and lipid biomarker analyses identified microorganisms virtually missed by DNA sequencing, such as *Cyanobacteria*. The most recalcitrant lipidic hydrocarbons may also suggest the presence of mosses and/or higher plant remnants, either from current material transported by wind from other locations in Antarctica or from ancient plant debris when the climate in Antarctica was warmer (e.g., Mid-Miocene or Eocene). The combination of the three biomolecule analyses allowed a comprehensive taxonomic and metabolic reconstruction of the old microbial mat before desiccation, elucidating oxygenic and anoxygenic photosynthesis, nitrogen fixation, nitrification, denitrification, sulfur reduction and oxidation, and methanogenesis. While relying on individual biomolecules leads to partial taxonomic and metabolic reconstructions, merging the taxonomic specificity and preservation capacity of DNA, proteins and lipidic biomolecules result in a potent forensic alternative to achieve a more comprehensive view of ancient scenarios.

## Data Availability Statement

Raw DNA sequence reads were deposited at the NCBI Sequence Read Archive (SRA) under the BioProject ID PRJNA707427. The protein data set can be found in Supplementary Table 1.

## Author Contributions

ML and VP conceived and designed the study with contributions from LS-G. AQ conducted the sampling. ML and AQ performed the growth assays. LS-G and ML performed the extraction, analysis and interpretation of the lipid biomarkers. ML performed the extraction, analysis and interpretation of DNA and proteins, and conducted DNA sequencing analysis together with MF-M. DC performed isotope analysis and interpreted the results together with LS-G. AQ and EC-S performed microscopy analysis and interpretation. AQ contributed to contextualizing the results in the McMurdo Ice Shelf ecosystem. ML performed the biological and functional reconstruction of the microbial mat and wrote the manuscript. All authors contributed to the discussion and editing of the manuscript and approved the submitted version.

## Funding

This research has been funded by the Spanish Ministry of Science and Innovation/State Agency of Research MCIN/AEI/10.13039/501100011033 and ERDF “A way of making Europe” through the grants RTI2018-094368-B-I00, CTM2016-79741-R and MDM-2017-0737 (Unidad de Excelencia Maria de Maeztu - Centro de Astrobiología (INTA-CSIC)). ML was supported by a Juan de la Cierva postdoctoral grant FJC2018-037246-I and by a postdoctoral grant from the European Union Youth Employment Initiative PEJD-2017-POST/TIC-4119 funded by AEI/10.13039/501100011033 and ESF “Investing in your future.” LS-G and DC were supported by a Ramón y Cajal grants RYC2018-023943-I and RYC-2014-19446, respectively, funded by MCIN/AEI/10.13039/501100011033 and ESF “Investing in your future.”

## Conflict of Interest

The authors declare that the research was conducted in the absence of any commercial or financial relationships that could be construed as a potential conflict of interest.

## Publisher’s Note

All claims expressed in this article are solely those of the authors and do not necessarily represent those of their affiliated organizations, or those of the publisher, the editors and the reviewers. Any product that may be evaluated in this article, or claim that may be made by its manufacturer, is not guaranteed or endorsed by the publisher.

## Footnotes

^1^ https://www.kegg.jp/kegg/tool/conv_id.html
