# Supplementary material for: Comprehensive Metabolic and Taxonomic Reconstruction of an Ancient Microbial Mat From the McMurdo Ice Shelf (Antarctica) by Integrating Genetic, Metaproteomic and Lipid Biomarker Analyses
Source: Front Microbiol. 2022 Apr 12;13:799360. doi: 10.3389/fmicb.2022.799360 (PMC9345047; doi:10.3389/fmicb.2022.799360)
Supplement: Supplementary file 1 [file Data_Sheet_1.pdf]

SUPPLEMENTARY MATERIAL

Comprehensive metabolic and taxonomic reconstruction of an ancient microbial mat from the McMurdo Ice Shelf (Antarctica) by integrating genetic, metaproteomic and lipid biomarker analyses

María Ángeles Lezcano[1*], Laura Sánchez-García[1], Antonio Quesada[2], Daniel Carrizo[1], Miguel Ángel Fernández-Martínez[3], Erika Cavalcante-Silva[2], Víctor Parro[1]

[1] Centro de Astrobiología (CAB), CSIC-INTA, Carretera de Ajalvir, km 4, 28850, Madrid, Spain
[2] Departamento de Biología, C. Darwin 2, Universidad Autónoma de Madrid, 28049, Madrid, Spain
[3] Department of Natural Resource Sciences, McGill University, 21,111 Lakeshore Rd., Ste. Anne de Bellevue, Quebec, Canada

***Correspondence:**

María Ángeles Lezcano
e-mail: mangeles.lezcano@gmail.com, Telephone: +34 915201936

Table of contents

Text 1. Supplementary material and methods.

Text 2. Supplementary discussion on the taxonomic specificity of proteins.

Text 3. Supplementary discussion on the identification of metabolisms in the microbial mat based on the presence of specific taxa.

Table 1. List of proteins in the microbial mat and negative samples (separated Excel file).

Figure 1. Map of the MIS Ponds and the location of the desiccated microbial mat of study at the time of collection during the austral summer of 1996.

Figure 2. Concentration of extracellular DNA and proteins (soluble in water) in the desiccated microbial mat.

Figure 3. Composition of the negative control of protein extraction and analysis.

Figure 4. Molecular distribution patterns of the three major lipid families.

Figure 5. Stable-carbon isotopic composition ($\delta^{13}C$) of *n*-alkanoic acids in the microbial mat.

Figure 6. Proportion of metabolic pathways in the desiccated microbial mat that belongs to "Other KEGG categories".

Text 1. Supplementary material and methods

Genomic DNA extraction. The pellet was divided into two subsamples to increase extraction yield and was extracted using the DNeasy PowerBiofilm kit (QIAGEN, Hilden, Germany) following the manufacturer's instructions with several modifications in the cell disruption step. Subsamples were introduced in Bead Tubes with 450 µL of the first solution buffer (MBL), then introduced in liquid nitrogen for 30 s and thawed at 40ºC, repeating the freeze-thaw cycle four times. During the bead beat step suggested by the kit, cells were disrupted in a homogenizer (FastPrep-24 5G, MP Biomedicals, Santa Ana, CA, USA) with two cycles at 60 $m \cdot s^{-1}$ for 40 s. Finally, subsample extracts were combined in a single MB Spin Column for DNA clean-up steps following the manufacturer's instructions.

PCR amplification and Illumina MiSeq sequencing. A first PCR was performed with a Q5 Hot Start High-Fidelity DNA Polymerase kit (New England Biolabs, Massachusetts, USA). The bacterial 16S rRNA gene was amplified using the primer pair 341-F/805-R (Herlemann et al., 2011), the archaeal 16S rRNA gene was amplified using the primer pair Arch1F/Arch1R (Cruaud et al., 2014) and the eukaryotic 18S rRNA gene was amplified using the primer pair 563F/1132R (Hugerth et al., 2014). The PCR for bacteria was performed with the following thermocycling conditions: 20 cycles of 98ºC for 10 s, 50ºC for 20s, and 72º for 20ºC, with a final extension step at 72ºC for 2 min. The PCR for archaea was performed at identical conditions except for 23 cycles instead of 20, and an annealing temperature of 48ºC. The PCR for eukarya was also performed at identical conditions except for 25 cycles instead of 20, and an annealing temperature of 54ºC to reduce amplification of an unspecific band at ~524 bp. A second PCR with 13 cycles was performed for all amplicons with the Q5 Hot Start High-Fidelity DNA Polymerase kit (New England Biolabs) to insert sample-specific barcodes and the Illumina adapter sequences (5'-AATGATACGGCGACCACCGAGATCTACACTGACGACATGGTTCTACA-3' and 5'-CAAGCAGAAGACGGCATACGAGAT-[10 nucleotides barcode]-TACGGTAGCAGAGACTTGGTCT-3') (Fluidigm, San Francisco, CA, USA). Final amplicons were validated and quantified with a 2100 Bioanalyzer (Agilent, Santa Clara, CA, USA).

Protein extraction. Pellets were extracted with sodium dodecyl sulfate (SDS) lysis and precipitated with trichloroacetic acid (TCA) following Hultman et al. (2015) with several modifications. Each sample was suspended in 7 mL of SDS lysis buffer (4 %, 100 mM Tris-HCl, pH 8.0) in Falcon tubes, boiled for 15 min, and pulse-sonicated (10 s on and 5 s off) for 5 min at 30 % amplitude. To assure cell disruption, boiling and sonication were repeated once. Samples were centrifuged at 21,000 x g for 15 min at 4 ºC, and supernatants containing the cell lysate were transferred to a fresh tube. Sample extracts were incubated with 20% TCA overnight at -20 ºC to precipitate the proteins and were centrifuged at 21,000 x g for 40 min at 4 ºC to discard supernatants. Protein pellets were washed with 1 mL of chilled acetone, vortexed, and centrifuged, repeating the process

three times. In the last wash, protein pellets were air-dried until acetone evaporated and then dissolved in urea 8M.

Protein analysis. Total protein biomass was digested in-gel with trypsin. Proteins were reduced with 10 mM DTT, alkylated with 55 mM iodoacetamide and digested with 4 $\mu g \cdot \mu L^{-1}$ of recombinant bovine trypsin (Roche Molecular Biochemicals) (1/25 w/w) overnight at 37ºC. Peptides were vacuum-dried by centrifugation and resuspended in 2% acetonitrile and 0.1% formic acid for analysis. Desalted protein digests were analyzed using a nano Easy-nLC 1000 system (Thermo Scientific) coupled to a high-resolution Q-Exactive HF hybrid quadrupole-Orbitrap mass spectrometer (Thermo Scientific). Samples were loaded onto an Acclaim PepMap 100 pre-column (20mm x 75 µm ID, 3 µm C18 with 100 Å pore size, Thermo Scientific) and then separated using an Easy-spray Column (500 mm x 75 µm ID, 2 µm C18 with 100 Å pore size, Thermo Scientific) with an integrated spray tip and a flow rate of 250 $nL \cdot min^{-1}$. The mobile phase consisted of 2% acetonitrile and 0.1% formic acid in water (A) and 0.1% formic acid in acetonitrile (B). Peptides were eluted using a gradient profile that started from 2% to 35% B for 150 min, increased to 45% B for 10 min, and changed to more than 95% B for 10 min. Data acquisition was performed using an ion spray voltage of 1.9 Kv and an ion transfer temperature of 270 ºC. Peptides were detected in positive ion mode and full scan mode for a mass range of 350-1800 Da.

Compound-specific isotope analysis. The carbon isotopic composition of individual lipids compounds was determined by coupling the gas chromatograph-mass spectrometer (Trace GC 1310 ultra and ISQ QD-MS, Thermo Fisher Scientific) to the isotope-ratio mass spectrometry system (MAT 253 IRMS, Thermo Fisher Scientific). For the gas chromatography analysis (GC), we used a TG-5MS column (30 m length, 0.25 mm inner diameter, with a thickness of 0.25 µm, Thermo Scientific) and the oven temperature was set to increase from 70 °C to 130 °C at 20 $°C \cdot min^{-1}$ and to 300 °C at 10 $°C \cdot min^{-1}$ (held for 15 min). For the analysis with isotope-ratio mass spectrometry (IRMS), conditions were: electron ionization 100 eV, Faraday cup collectors m/z 44, 45, and 46, and temperature of the CuO/NiO combustion interface at 1000 °C. The samples were injected in a PTV injector in splitless mode, with an inlet temperature of 250 ºC. Helium was used as carrier gas at a constant flow of 1.1 $ml \cdot min^{-1}$. For the alkanoic acids, the $\delta^{13}C$ data were calculated from the FAME values, correcting them for the one carbon atom added in the methanolysis (Abrajano et al., 1994).

Text 2. Supplementary discussion on the taxonomic specificity of proteins

Traditionally, metaproteomics was used for assessing the metabolism of microbial communities. However, there is an increasing interest in metaproteomics for characterising the structure of microbial communities and quantifying the relative abundance of taxa (Kleiner et al., 2017; Kleiner, 2019). Here, we complement and compare the microbial community profile of an ancient microbial mat based on SSU rRNA gene sequencing and metaproteomics analyses. We performed this comparison mainly at the phylum and order level. Therefore, species-level comparisons were avoided due to possible protein misidentification, addressing the following issues:

i) **Protein inference.** In proteomics, proteins are identified by matching peptide sequences from mass spectrometry to specific proteins. Occasionally, the same peptide sequence can match different proteins, which may lead to ambiguous protein identification. Particularly when conducting metaproteomics, multiple species with proteins sequences may also share peptides with protein sequences from other species. This may result in certain proteins with incorrect species annotation (Kleiner et al., 2017).
ii) **Protein databases.** Currently, protein databases are comparatively smaller than those of DNA (e.g., 16S and 18S rRNA genes), and are relatively enriched in complete or nearly complete proteomes of model organisms, which may also lead to incorrect species annotation (Hendy et al., 2018).

Despite accurate species-level assignments has been successfully done by the construction of specific databases and metagenomics workflows (Kleiner et al., 2017), in our study, we used entries of the Swiss-Prot database without taxonomic restrictions. Therefore, for a conservative approach, we investigated the taxonomic profile at the phylum, order and, occasionally, at the genus level (Supplementary Text 3), which are taxonomic resolutions that can overcome the aforementioned limitations.

Text 3. Supplementary discussion on the identification of biological sources and metabolisms in the microbial mat based on the presence of specific taxa.

Identification of biological sources and metabolisms in the desiccated microbial mat was based on the detection of specific taxa (at the phylum, order, and/or genus level) with 16S and 18S rRNA gene metabarcoding, metaproteomics, and/or lipid biomarker analysis. Two inputs of biological sources were considered in the microbial mat: autochthonous, belonging to the microbial mat itself, and allochthonous, built-in from the surroundings (e.g., lipid signatures potentially from mosses and higher plants). The taxa identified as potentially involved in specific microbial metabolisms are those related to the indigenous microorganisms in the microbial mat and are detailed below. Additionally, a section with the identification of potentially allochthonous biological sources related to specific lipid compounds was also included.

Oxygenic photosynthesis:

- DNA: *Chlorophyta* (*Chlorophyceae)* and *Ochrophyta* (*Chromulinales*) (Gantt, 2011)
- Proteins: *Cyanobacteria* (*Nostocales,* mostly *Nostoc*; and *Synechococcales,* mostly *Synechococcus*), *Chlorophyta* (*Chlamydomonadales, Chlorellales, Mamiellales, Nephroselmidales* and *Oltmannsiellopsidales*), *Ochrophyta* (*Naviculales)* and *Haptophyta* (*Isochrysidales*) (Gantt, 2011)
- Lipids biomarkers:
    - Non-polar fraction - alkanes: *n*-C_{17}, 7-methyl C_{16}, 7-methyl C_{17}, 7-methyl C_{18} and 7-methyl C_{19} indicative of cyanobacteria (Gelpi et al., 1970; Shiea et al., 1990; Rontani and Volkman, 2005; Allen et al., 2010). Pristane and phytane, majorly transformation products of phytol, thus indicative of chlorophyll-bearing organisms (Rontani and Volkman, 2003).
    - Acidic fraction - alkanoic acids: unsaturated moieties such as 16:1(ω7) and 18:1(ω9), suggesting the presence of cyanobacteria and/or microalgae (Allen et al., 2010; Pagès et al., 2015).
    - Polar fraction - alkanols: Phytol, indicative of chlorophyll-bearing organisms (Brocks and Summons, 2003). Dinosterol, indicative of diatoms (Volkman, 2003), and fucosterol, campesterol, β-sitosterol, and stigmasterol, suggesting the presence of microalgae (Martin-Creuzburg and Merkel, 2016; Randhir et al., 2020).

Carbon oxidation:

- DNA: prokaryotes and eukaryotes (e.g. *Chytridiomycota*, *Ascomycota*, *Ciliophora,* and *Cercozoa*).
- Proteins: prokaryotes and eukaryotes
- Lipid biomarkers: eukaryotes (e.g. ergostanol, suggesting the presence of fungi (Weete et al., 2010)).

Nitrogen fixation:

- DNA: *Rhizobiales* (Rascio and La Rocca, 2013), *Clostridiales (Clostridium* (Rascio and La Rocca, 2013)).
- Proteins: *Rhizobiales, Clostridiales* (*Clostridium*), *Nostocales* (Sukenik et al., 2009), *Frankiales* (*Frankia* (Sellstedt and Richau, 2013)), *Campylobacterales* (*Arcobacter* (Pati et al., 2010)), *Rhodospirillales* (*Gluconacetobacter* (Van Dommelen and Vanderleyden, 2007)).

Nitrification:

- DNA: *Nitrosomonadales* (*Nitrospira* (Prosser, 2007))
- Proteins: *Nitrosomonadales* (*Nitrosomonas* (Prosser, 2007)), *Chromatiales* (*Nitrosococcus* (Prosser, 2007)).

Denitrification:

- DNA: *Actinomycetales* (*Tetrasphaera* (Marques et al., 2018)).
- Proteins: *Nitrosomonadales* (*Thiobacillus* (Friedrich et al., 2005)).

Anoxygenic photosynthesis:

- DNA: *Burkholderiales* (*Rhodoferax* (Madigan et al., 2000)).
- Proteins: *Chromatiales* (Imhoff et al., 2005), *Rhodobacterales (Dinoroseobacter* (Biebl et al., 2005), *Rhodobacter* (Imhoff et al., 2005)), *Sphingomonadales* (*Erythrobacter* (Sato-Takabe et al., 2012)).

Sulfur oxidation:

- DNA: *Burkholderiales* (*Rhodoferax* (Madigan et al., 2000)).
- Proteins: *Nitrosomonadales* (*Thiobacillus* (Friedrich et al., 2005)), *Campylobacterales* (*Arcobacter* (Sievert et al., 2007)), *Chromatiales* (*Halothiobacillus* (Kelly and Wood, 2015)).

Sulfur reduction (elemental sulfur and sulfate reducers):

- DNA: *Clostridiales* (*Desulfosporosinus* (Ramamoorthy et al., 2006), *Clostridium* (Sallam and Steinbuchel, 2009)), *Desulfobacterales* (*Desulfocapsa* (Janssen et al., 1996)), *Desulfuromonadales* (*Desulfuromonas* (Fenchel et al., 2012), *Geobacter* (Fenchel et al., 2012)).
- Proteins: *Clostridiales* (*Desulfitobacterium* (Villemur et al., 2006), *Clostridium*), *Desulfobacterales* (*Desulfotalea* (Rabus et al., 2004)), *Desulfuromonadales* (*Geobacter*).
- Lipid biomarkers:
  - Alkanoic acids: i/a-C_{15}, i/a-C_{16}, i/a-C_{17}, i/a-C_{18}, often associated with sulfate-reducing bacteria (Taylor and Parkes, 1983; Kaneda, 1991).

Methanogenesis:

- DNA: *Methanomicrobiales* (Fenchel et al., 2012), *Methanosarcinales* (Fenchel et al., 2012).
- Proteins: *Methanomicrobiales*, *Methanosarcinales*.
- Lipids biomarkers:
    - Alkanes: phytane, potentially indicative of methanogenic archaea when it comes from the degradation of archaeol (diphytanylglycerol) under anoxic conditions (Brocks and Summons, 2003).

Allochthonous sources based on lipid biomarkers:

- Alkanes: odd HMW alkanes can be produced by mosses (e.g. *n*-C_{23} and *n*-C_{25} (Nott et al., 2000; Pancost et al., 2002), macrophytes (e.g. *n*-C_{23} and *n*-C_{25} (Ficken et al., 2000; Mead et al., 2005) or higher plants (e.g. *n*-C_{27} and *n*-C_{29} (Eglinton and Hamilton, 1967; Hedges and Prahl, 1993)).
- Alkanoic acids: even HMW alkanoic acids such as 24:0, 26:0, and 28:0 may be indicative of higher plants (Eglinton and Hamilton, 1967; Hedges and Prahl, 1993) or microbial sources (Naraoka and Ishiwatari, 2000; Chen et al., 2019), depending on their $\delta^{13}C$ signatures relatively depleted (plants or microalgae) or enriched (heterotrophic microorganisms) in ^{13}C (Chen et al., 2019).
- Alkanols: even HMW alkanols (e.g. *n*-C_{24}, *n*-C_{26} and *n*-C_{28}) can stem from higher plants (Eglinton and Hamilton, 1967). In addition, phytosterols such as fucosterol, campesterol, β-sitosterol, and stigmasterol may be produced, apart from micro-/macro-algae (Martin-Creuzburg and Merkel, 2016; Taipale et al., 2016; Pereira et al., 2017; Randhir et al., 2020), from higher plants (Volkman, 1986).

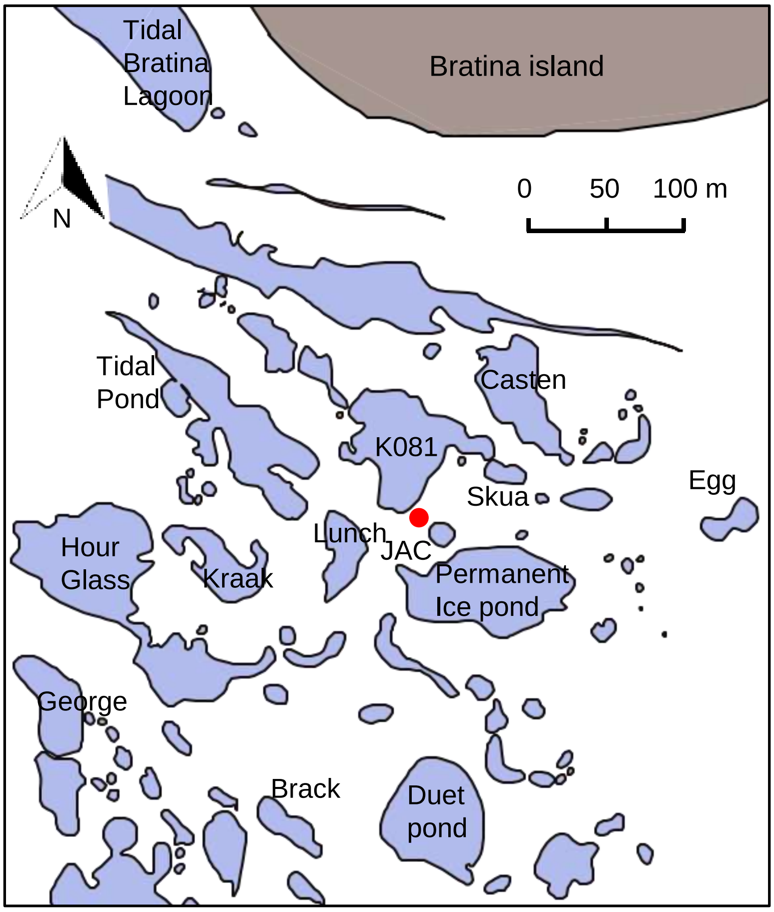

Figure 1. Map of the MIS Ponds, based on Archer et al. (2014) and Jackson et al. (2021), and the location (indicated as a red dot) of the desiccated microbial mat at the time of collection during the austral summer of 1996.

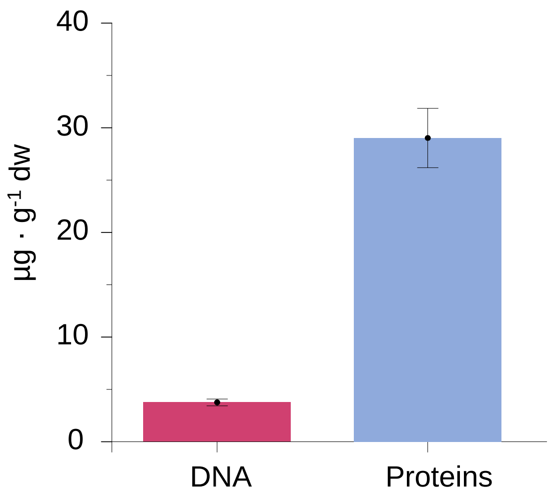

Figure 2. Concentration of extracellular DNA and proteins (soluble in water) in the desiccated microbial mat (μg·g^{-1} of dry weight). Error bars are the standard deviation of triplicates.

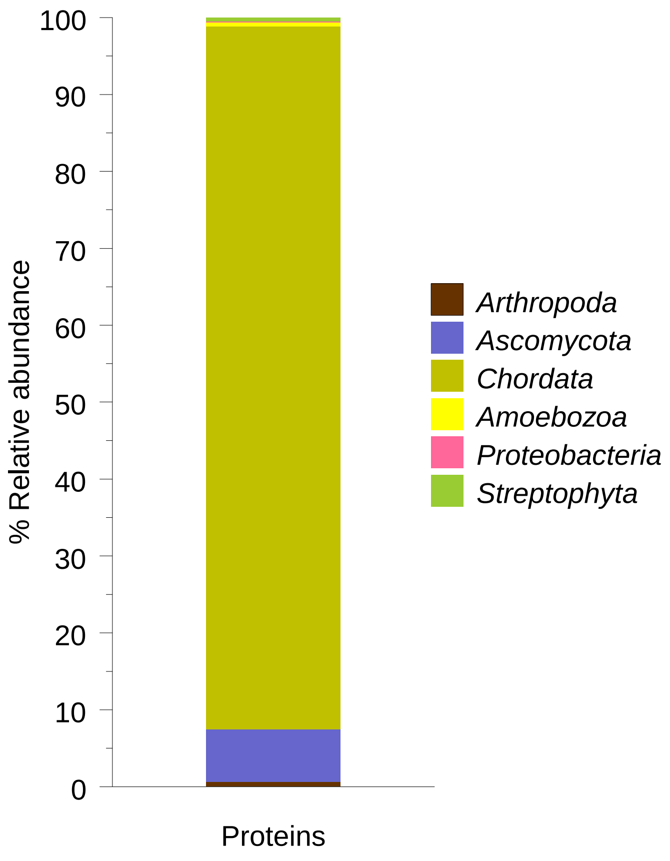

Figure 3. Biological composition of the negative control during protein extraction and analysis calculated based on the normalized spectral abundance factor (NSAF) annotated per cent (more details in Materials and methods).

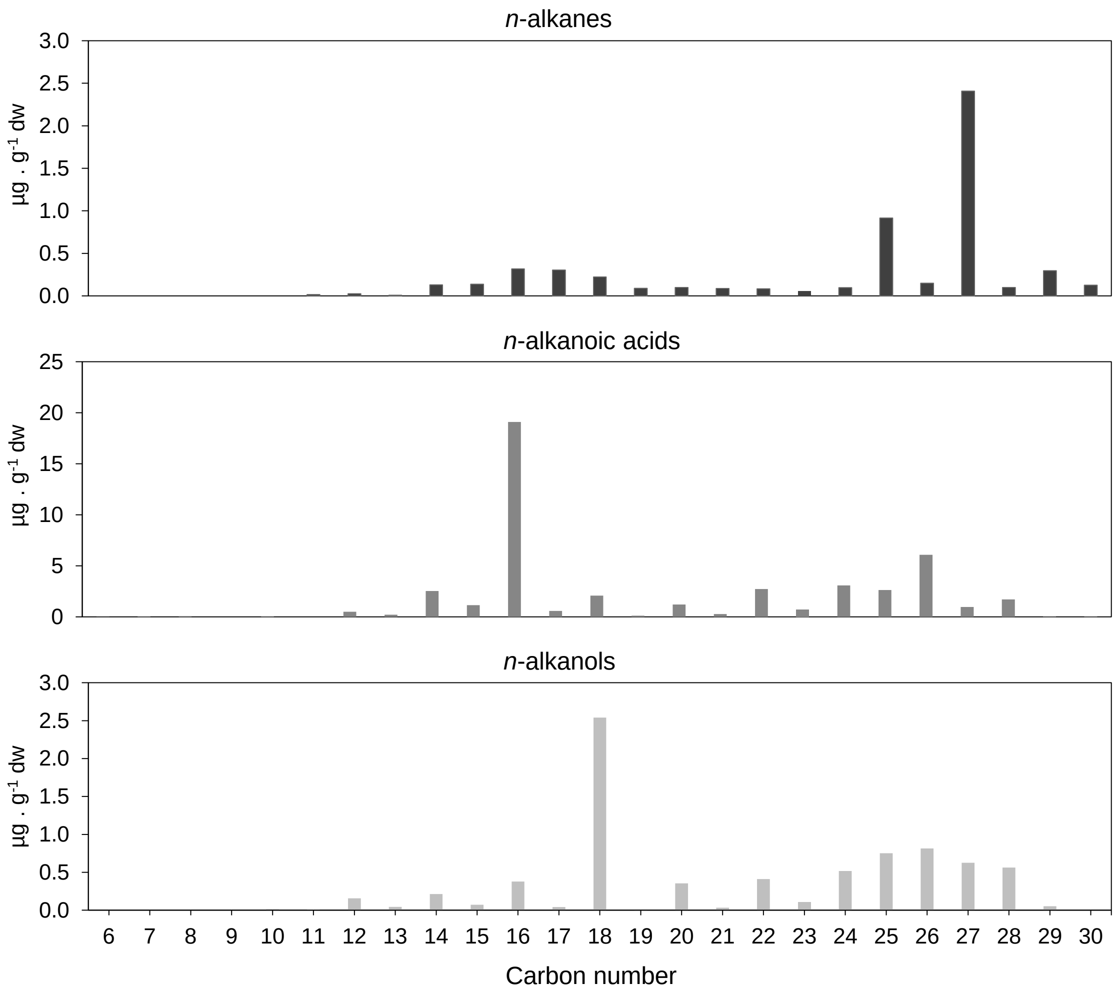

Figure 4. Molecular distribution patterns of the three major lipid families (*n*-alkanes, *n*-alkanoic acids and *n*-alkanols) in the ancient microbial mat after integration of the peak areas (Figure 6A, B and C) and conversion to concentration units (µg of compound per g of sample, as dry weight).

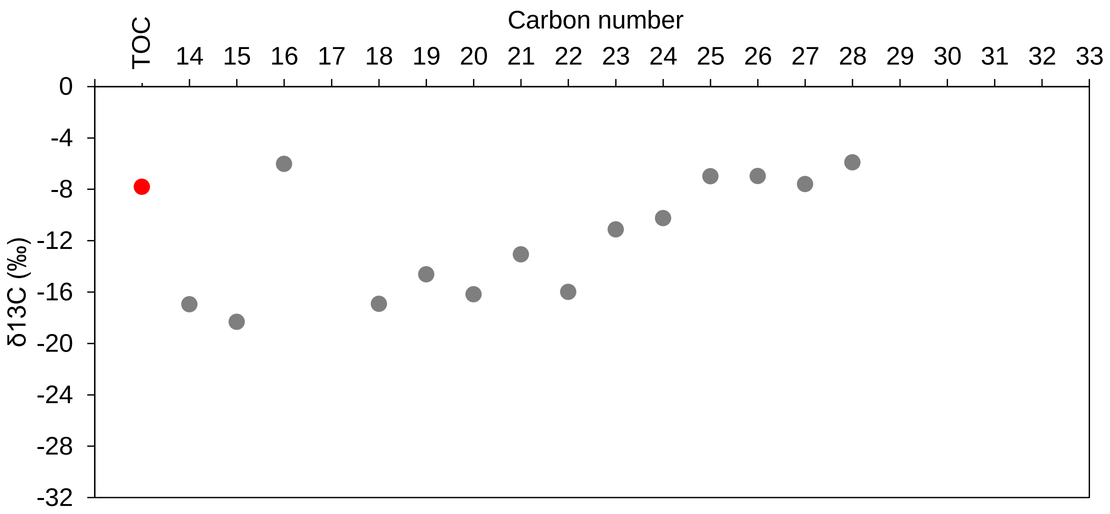

Figure 5. Stable-carbon isotopic composition ($\delta^{13}C$) of the *n*-alkanoic acids in the ancient microbial mat. The isotopic composition of the bulk biomass (TOC) was also represented as a red circle. The standard deviation of triplicates of the $\delta^{13}C_{TOC}$ is hidden behind the circle.

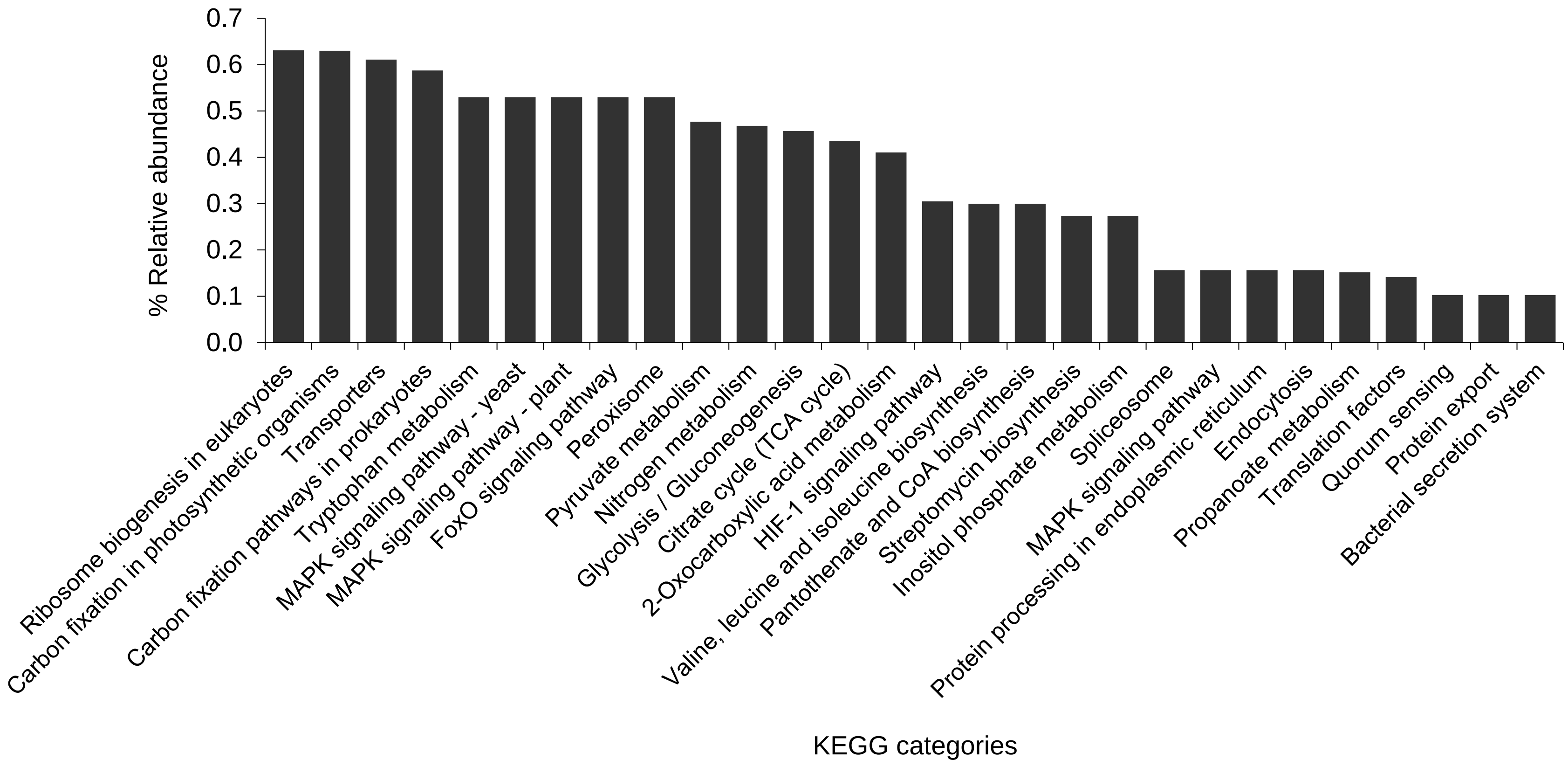

Figure 6. Proportion of metabolic pathways in the desiccated microbial mat that belongs to "Other KEGG categories" in Figure 8 from the main text. The relative abundance of KEGG categories was calculated based on the sum of SAFs of the proteins that are annotated in each KEGG category.

References

Abrajano, T. A., Murphy, D. E., Fang, J., Comet, P., and Brooks, J. M. (1994). 13C/12C ratios in individual fatty acids of marine mytilids with and without bacterial symbionts. *Org. Geochem.* 21, 611–617. doi:10.1016/0146-6380(94)90007-8.

Allen, M. A., Neilan, B. A., Burns, B. P., Jahnke, L. L., and Summons, R. E. (2010). Lipid biomarkers in Hamelin Pool microbial mats and stromatolites. *Org. Geochem.* 41, 1207–1218. doi:10.1016/j.orggeochem.2010.07.007.

Archer, S. D. J., McDonald, I. R., Herbold, C. W., and Cary, S. C. (2014). Characterisation of bacterioplankton communities in the meltwater ponds of Bratina Island, Victoria Land, Antarctica. *FEMS Microbiol. Ecol.* 89, 451–464. doi:10.1111/1574-6941.12358.

Biebl, H., Allgaier, M., Tindall, B. J., Koblizek, M., Lünsdorf, H., Pukall, R., et al. (2005). Dinoroseobacter shibae gen. nov., sp. nov., a new aerobic phototrophic bacterium isolated from dinoflagellates. *Int. J. Syst. Evol. Microbiol.* 55, 1089–1096. doi:10.1099/ijs.0.63511-0.

Brocks, J. J., and Summons, R. E. (2003). "Sedimentary Hydrocarbons, Biomarkers for Early Life," in *Treatise on Geochemistry* (Elsevier), 63–115. doi:10.1016/B0-08-043751-6/08127-5.

Chen, X., Liu, X., Wei, Y., and Huang, Y. (2019). Production of long-chain n-alkyl lipids by heterotrophic microbes: New evidence from Antarctic lakes. *Org. Geochem.* 138, 103909. doi:10.1016/j.orggeochem.2019.103909.

Cruaud, P., Vigneron, A., Lucchetti-Miganeh, C., Ciron, P. E., Godfroy, A., and Cambon-Bonavita, M. A. (2014). Influence of DNA extraction method, 16S rRNA targeted hypervariable regions, and sample origin on microbial diversity detected by 454 pyrosequencing in marine chemosynthetic ecosystems. *Appl. Environ. Microbiol.* 80, 4626–4639. doi:10.1128/AEM.00592-14.

Eglinton, G., and Hamilton, R. J. (1967). Leaf Epicuticular Waxes. *Science (80-.).* 156, 1322–1335. doi:10.1126/science.156.3780.1322.

Fenchel, T., King, G. M., and Blackburn, T. H. (2012). "Bacterial Metabolism," in *Bacterial Biogeochemistry* (Elsevier), 1–34. doi:10.1016/B978-0-12-415836-8.00001-3.

Ficken, K. ., Li, B., Swain, D. ., and Eglinton, G. (2000). An n-alkane proxy for the sedimentary input of submerged/floating freshwater aquatic macrophytes. *Org. Geochem.* 31, 745–749. doi:10.1016/S0146-6380(00)00081-4.

Friedrich, C. G., Bardischewsky, F., Rother, D., Quentmeier, A., and Fischer, J. (2005). Prokaryotic sulfur oxidation. *Curr. Opin. Microbiol.* 8, 253–259. doi:10.1016/j.mib.2005.04.005.

Gantt, E. (2011). Oxygenic photosynthesis and the distribution of chloroplasts. *Photosynth. Res.* 107, 1–6. doi:10.1007/s11120-010-9605-1.

Gelpi, E., Schneider, H., Mann, J., and Oró, J. (1970). Hydrocarbons of geochemical significance in microscopic algae. *Phytochemistry* 9, 603–612. doi:10.1016/S0031-9422(00)85700-3.

Hedges, J. I., and Prahl, F. G. (1993). "Early Diagenesis: Consequences for Applications of Molecular Biomarkers," in *Organic Geochemistry. Topics in Geobiology*, eds. M. H. Engel and S. A. Macko (Boston, MA: Springer), 237–253. doi:10.1007/978-1-4615-2890-6_11.

Hendy, J., Welker, F., Demarchi, B., Speller, C., Warinner, C., and Collins, M. J. (2018).

A guide to ancient protein studies. *Nat. Ecol. Evol.* 2, 791–799. doi:10.1038/s41559-018-0510-x.
Herlemann, D. P. R., Labrenz, M., Jürgens, K., Bertilsson, S., Waniek, J. J., and Andersson, A. F. (2011). Transitions in bacterial communities along the 2000 km salinity gradient of the Baltic Sea. *ISME J.* 5, 1571–1579. doi:10.1038/ismej.2011.41.
Hugerth, L. W., Muller, E. E. L., Hu, Y. O. O., Lebrun, L. A. M., Roume, H., Lundin, D., et al. (2014). Systematic design of 18S rRNA gene primers for determining eukaryotic diversity in microbial consortia. *PLoS One* 9, e95567. doi:10.1371/journal.pone.0095567.
Hultman, J., Waldrop, M. P., Mackelprang, R., David, M. M., McFarland, J., Blazewicz, S. J., et al. (2015). Multi-omics of permafrost, active layer and thermokarst bog soil microbiomes. *Nature* 521, 208–212. doi:10.1038/nature14238.
Imhoff, J. F., Hiraishi, A., and Süling, J. (2005). "Anoxygenic Phototrophic Purple Bacteria," in *Bergey's Manual® of Systematic Bacteriology* (Boston, MA: Springer US), 119–132. doi:10.1007/0-387-28021-9_15.
Jackson, E. E., Hawes, I., and Jungblut, A. D. (2021). 16S rRNA gene and 18S rRNA gene diversity in microbial mat communities in meltwater ponds on the McMurdo Ice Shelf, Antarctica. *Polar Biol.* doi:10.1007/s00300-021-02843-2.
Janssen, P. H., Schuhmann, A., Bak, F., and Liesack, W. (1996). Disproportionation of inorganic sulfur compounds by the sulfate-reducing bacterium Desulfocapsa thiozymogenes gen. nov., sp. nov. *Arch. Microbiol.* 166, 184–192. doi:10.1007/s002030050374.
Kaneda, T. (1991). Iso- and anteiso-fatty acids in bacteria: biosynthesis, function, and taxonomic significance. *Microbiol. Rev.* 55, 288–302. doi:10.1128/mr.55.2.288-302.1991.
Kelly, D. P., and Wood, A. P. (2015). "Halothiobacillus," in *Bergey's Manual of Systematics of Archaea and Bacteria* (Wiley), 1–3. doi:10.1002/9781118960608.gbm01133.
Kleiner, M. (2019). Metaproteomics: Much More than Measuring Gene Expression in Microbial Communities. *mSystems* 4, 1–6. doi:10.1128/mSystems.00115-19.
Kleiner, M., Thorson, E., Sharp, C. E., Dong, X., Liu, D., Li, C., et al. (2017). Assessing species biomass contributions in microbial communities via metaproteomics. *Nat. Commun.* 8, 1558. doi:10.1038/s41467-017-01544-x.
Madigan, M. T., Jung, D. O., Woese, C. R., and Achenbach, L. A. (2000). Rhodoferax antarcticus sp. nov., a moderately psychrophilic purple nonsulfur bacterium isolated from an Antarctic microbial mat. *Arch. Microbiol.* 173, 269–277. doi:10.1007/s002030000140.
Marques, R., Ribera-Guardia, A., Santos, J., Carvalho, G., Reis, M. A. M., Pijuan, M., et al. (2018). Denitrifying capabilities of Tetrasphaera and their contribution towards nitrous oxide production in enhanced biological phosphorus removal processes. *Water Res.* 137, 262–272. doi:10.1016/j.watres.2018.03.010.
Martin-Creuzburg, D., and Merkel, P. (2016). Sterols of freshwater microalgae: potential implications for zooplankton nutrition. *J. Plankton Res.* 38, 865–877. doi:10.1093/plankt/fbw034.
Mead, R., Xu, Y., Chong, J., and Jaffé, R. (2005). Sediment and soil organic matter source assessment as revealed by the molecular distribution and carbon isotopic composition of n-alkanes. *Org. Geochem.* 36, 363–370. doi:10.1016/j.orggeochem.2004.10.003.
Naraoka, H., and Ishiwatari, R. (2000). Molecular and isotopic abundances of long-chain

n-fatty acids in open marine sediments of the western North Pacific. *Chem. Geol.* 165, 23–36. doi:10.1016/S0009-2541(99)00159-X.

Nott, C. J., Xie, S., Avsejs, L. A., Maddy, D., Chambers, F. M., and Evershed, R. P. (2000). n-Alkane distributions in ombrotrophic mires as indicators of vegetation change related to climatic variation. *Org. Geochem.* 31, 231–235. doi:10.1016/S0146-6380(99)00153-9.

Pagès, A., Grice, K., Welsh, D. T., Teasdale, P. T., Van Kranendonk, M. J., and Greenwood, P. (2015). Lipid Biomarker and Isotopic Study of Community Distribution and Biomarker Preservation in a Laminated Microbial Mat from Shark Bay, Western Australia. *Microb. Ecol.* 70, 459–472. doi:10.1007/s00248-015-0598-3.

Pancost, R. D., Baas, M., van Geel, B., and Sinninghe Damsté, J. S. (2002). Biomarkers as proxies for plant inputs to peats: an example from a sub-boreal ombrotrophic bog. *Org. Geochem.* 33, 675–690. doi:10.1016/S0146-6380(02)00048-7.

Pati, A., Gronow, S., Lapidus, A., Copeland, A., Glavina Del Rio, T., Nolan, M., et al. (2010). Complete genome sequence of Arcobacter nitrofigilis type strain (CIT). *Stand. Genomic Sci.* 2, 300–308. doi:10.4056/sigs.912121.

Pereira, C. M. P., Nunes, C. F. P., Zambotti-Villela, L., Streit, N. M., Dias, D., Pinto, E., et al. (2017). Extraction of sterols in brown macroalgae from Antarctica and their identification by liquid chromatography coupled with tandem mass spectrometry. *J. Appl. Phycol.* 29, 751–757. doi:10.1007/s10811-016-0905-5.

Prosser, J. I. (2007). “The Ecology of Nitrifying Bacteria,” in *Biology of the Nitrogen Cycle* (Elsevier), 223–243. doi:10.1016/B978-044452857-5.50016-3.

Rabus, R., Ruepp, A., Frickey, T., Rattei, T., Fartmann, B., Stark, M., et al. (2004). The genome of Desulfotalea psychrophila, a sulfate-reducing bacterium from permanently cold Arctic sediments. *Environ. Microbiol.* 6, 887–902. doi:10.1111/j.1462-2920.2004.00665.x.

Ramamoorthy, S., Sass, H., Langner, H., Schumann, P., Kroppenstedt, R. M., Spring, S., et al. (2006). Desulfosporosinus lacus sp. nov., a sulfate-reducing bacterium isolated from pristine freshwater lake sediments. *Int. J. Syst. Evol. Microbiol.* 56, 2729–2736. doi:10.1099/ijs.0.63610-0.

Randhir, A., Laird, D. W., Maker, G., Trengove, R., and Moheimani, N. R. (2020). Microalgae: A potential sustainable commercial source of sterols. *Algal Res.* 46, 101772. doi:10.1016/j.algal.2019.101772.

Rascio, N., and La Rocca, N. (2013). “Biological Nitrogen Fixation,” in *Encyclopedia of Ecology* (Elsevier), 264–279. doi:10.1016/B978-0-444-63768-0.00685-5.

Rontani, J.-F., and Volkman, J. K. (2003). Phytol degradation products as biogeochemical tracers in aquatic environments. *Org. Geochem.* 34, 1–35. doi:10.1016/S0146-6380(02)00185-7.

Rontani, J.-F., and Volkman, J. K. (2005). Lipid characterization of coastal hypersaline cyanobacterial mats from the Camargue (France). *Org. Geochem.* 36, 251–272. doi:10.1016/j.orggeochem.2004.07.017.

Sallam, A., and Steinbuchel, A. (2009). Clostridium sulfidigenes sp. nov., a mesophilic, proteolytic, thiosulfate- and sulfur-reducing bacterium isolated from pond sediment. *Int. J. Syst. Evol. Microbiol.* 59, 1661–1665. doi:10.1099/ijs.0.004986-0.

Sato-Takabe, Y., Hamasaki, K., and Suzuki, K. (2012). Photosynthetic characteristics of marine aerobic anoxygenic phototrophic bacteria Roseobacter and Erythrobacter strains. *Arch. Microbiol.* 194, 331–341. doi:10.1007/s00203-011-0761-2.

Sellstedt, A., and Richau, K. H. (2013). Aspects of nitrogen-fixing Actinobacteria, in particular free-living and symbiotic Frankia. *FEMS Microbiol. Lett.* 342, 179–186.

doi:10.1111/1574-6968.12116.
Shiea, J., Brassell, S. C., and Ward, D. M. (1990). Mid-chain branched mono- and dimethyl alkanes in hot spring cyanobacterial mats: A direct biogenic source for branched alkanes in ancient sediments? *Org. Geochem.* 15, 223–231. doi:10.1016/0146-6380(90)90001-G.
Sievert, S. M., Wieringa, E. B. A., Wirsen, C. O., and Taylor, C. D. (2007). Growth and mechanism of filamentous-sulfur formation by Candidatus Arcobacter sulfidicus in opposing oxygen-sulfide gradients. *Environ. Microbiol.* 9, 271–276. doi:10.1111/j.1462-2920.2006.01156.x.
Sukenik, A., Zohary, T., and Padisák, J. (2009). "Cyanoprokaryota and Other Prokaryotic Algae," in *Encyclopedia of Inland Waters* (Elsevier), 138–148. doi:10.1016/B978-012370626-3.00133-2.
Taipale, S. J., Hiltunen, M., Vuorio, K., and Peltomaa, E. (2016). Suitability of Phytosterols Alongside Fatty Acids as Chemotaxonomic Biomarkers for Phytoplankton. *Front. Plant Sci.* 7. doi:10.3389/fpls.2016.00212.
Taylor, J., and Parkes, R. J. (1983). The Cellular Fatty Acids of the Sulphate-reducing Bacteria, Desulfobacter sp., Desulfobulbus sp. and Desulfovibrio desulfuricans. *Microbiology* 129, 3303–3309. doi:10.1099/00221287-129-11-3303.
Van Dommelen, A., and Vanderleyden, J. (2007). "Associative Nitrogen Fixation," in *Biology of the Nitrogen Cycle* (Elsevier), 179–192. doi:10.1016/B978-044452857-5.50013-8.
Villemur, R., Lanthier, M., Beaudet, R., and Lépine, F. (2006). The Desulfitobacterium genus. *FEMS Microbiol. Rev.* 30, 706–733. doi:10.1111/j.1574-6976.2006.00029.x.
Volkman, J. (2003). Sterols in microorganisms. *Appl. Microbiol. Biotechnol.* 60, 495–506. doi:10.1007/s00253-002-1172-8.
Volkman, J. K. (1986). A review of sterol markers for marine and terrigenous organic matter. *Org. Geochem.* 9, 83–99. doi:10.1016/0146-6380(86)90089-6.
Weete, J. D., Abril, M., and Blackwell, M. (2010). Phylogenetic Distribution of Fungal Sterols. *PLoS One* 5, e10899. doi:10.1371/journal.pone.0010899.
